# A ferroptosis-related signature predicts the clinical diagnosis and prognosis, and associates with the immune microenvironment of lung cancer

**DOI:** 10.1007/s12672-024-01032-x

**Published:** 2024-05-14

**Authors:** Hua Zhou, Xiaoting Zhou, Runying Zhu, Zhongquan Zhao, Kang Yang, Zhenghai Shen, Hongwen Sun

**Affiliations:** 1 Department of Oncology Radiotherapy, First Affiliated Hospital of Kunming Medical University, Kunming, 650032 Yunnan China; 2 Medical School, Kunming University of Science and Technology, Kunming, 650031 Yunnan China; 3 Department of Thoracic Surgery, First Affiliated Hospital of Kunming Medical University, No.295 Xichang Rd, Kunming, 650032 Yunnan China; 4 Department of Thoracic Surgery, Yunnan Cancer Hospital, Kunming, 650118 Yunnan China

**Keywords:** Lung cancer, Ferroptosis, Diagnosis, Prognosis, Tumor immune microenvironment

## Abstract

Targeting ferroptosis-related pathway is a potential strategy for treatment of lung cancer (LC). Consequently, exploration of ferroptosis-related markers is important for treating LC. We collected LC clinical data and mRNA expression profiles from TCGA and GEO database. Ferroptosis-related genes (FRGs) were obtained through FerrDB database. Expression analysis was performed to obtain differentially expressed FRGs. Diagnostic and prognostic models were constructed based on FRGs by LASSO regression, univariate, and multivariate Cox regression analysis, respectively. External verification cohorts GSE72094 and GSE157011 were used for validation. The interrelationship between prognostic risk scores based on FRGs and the tumor immune microenvironment was analyzed. Immunocytochemistry, Western blotting, and RT-qPCR detected the FRGs level. Eighteen FRGs were used for diagnostic models, 8 FRGs were used for prognostic models. The diagnostic model distinguished well between LC and normal samples in training and validation cohorts of TCGA. The prognostic models for TCGA, GSE72094, and GSE157011 cohorts significantly confirmed lower overall survival (OS) in high-risk group, which demonstrated excellent predictive properties of the survival model. Multivariate Cox regression analysis further confirmed risk score was an independent risk factor related with OS. Immunoassays revealed that in high-risk group, a significantly higher proportion of Macrophages_M0, Neutrophils, resting Natural killer cells and activated Mast cells and the level of B7H3, CD112, CD155, B7H5, and ICOSL were increased. In conclusion, diagnostic and prognostic models provided superior diagnostic and predictive power for LC and revealed a potential link between ferroptosis and TIME.

**Supplementary Information:**

The online version contains supplementary material available at 10.1007/s12672-024-01032-x.

## Highlights

1. Ferroptosis-related prognostic risk score, T stage, denovo tumors were prognostic risk factors of LC.
2. B cells, neutrophils, and CD^+^T cells differ between high and low risk group.
3. Ferroptosis-related prognostic risk score could well predict the 5-year survival rate of patients.
4. ACSL3, FADS2, GLS2, HSF1, PANX1, PHKG2, and VDAC2 levels in high-risk group were increased.
5. CDKN1A level was decreased in high-risk group.

**Supplementary Information:**

The online version contains supplementary material available at 10.1007/s12672-024-01032-x.

## Introduction

Lung cancer (LC) is one of the fastest growing malignant tumors in term of morbidity and mortality, and one of the most threatening to human health and life. According to statistics, LC patients accounted for 13% of all new diagnoses and 24% of all cancer deaths in 2019 [1]. The vast majority of patients diagnosed with non-small cell lung cancer (NSCLC), the most common form of LC and accounting for 85%, are already at advanced or distant stages [2, 3]. Despite the current advances in surgery, radiotherapy and immunotherapy, the 5-year survival rate is only 4–17% [1, 4]. In recent years, diagnosis and prognosis models have become increasingly abundant in medical research, and they provide a useful reference for cancer diagnosis and prediction of cancer recurrence or death [5, 6]. Therefore, this study aims to construct predictive models that provide potential key biomarkers for LC.

Ferroptosis was first proposed in 2012, and is a novel form of programmed cell death [7]. Unlike autophagy and apoptosis, ferroptosis is a type of iron- and reactive oxygen species (ROS)-dependent cell death, and its mainly characterized by cytological changes [8]. New evidences suggested that ferroptosis maybe an adaptive process that was essential for eliminating cancer-causing cells [9–11]. In LC, ferroptosis was first triggered using Erastin (an ferroptosis activator) in A549 cells with K-ras mutant [12]. Subsequently, Erastin was found to sensitize LC cells to the apoptosis-inducing agent cisplatin by inhibiting glutathione peroxidase to reduce glutathione in an ferroptosis manner [13]. Therefore, ferroptosis-related genes (FRGs) are highly likely to serve as biomarkers with great potential in diagnostic and prognostic models of LC.

Cancer biology and immunosurveillance are inextricably linked. In the process of tumor development, the complex tumor immune microenvironment (TIME) closely interacts with tumor cells and tumor stroma, which have an invaluable role in monitoring and preventing tumor growth [14]. A central link between cancer biology and TIME is the iron competition between tumor cells and the immune system [15]. Iron is closely associated with the regulation of innate and adaptive responses in TIME, particularly in T cells and macrophages [16]. Macrophages resident in tissues are the “gatekeepers” of iron homeostasis, which absorb, metabolize, store, and export iron to meet the needs of the surrounding cells [17]. In tumor immunity, iron is necessary for t cell proliferation and effector function [18]. Hence, it is important to explore the correlation between FRGs and TIME for the diagnosis and prognosis of LC.

To explore the role of FRGs in the clinical diagnosis and prognosis of LC, in current study, a comprehensive analysis of LC cohorts in the cancer genome atlas (TCGA) and gene expression omnibus (GEO) databases was performed by bioinformatics methods to identify FRGs that are closely associated with LC prognosis. In addition, a FRGs-based LC diagnosis and prognosis prediction model was constructed, and the relationship between FRGs and immune infiltration of LC was explored. Our diagnosis and prognosis model may enhance early diagnosis of LC and ameliorate personalized prognostic assessment.

## Materials and methods

### Acquisition of FRGs

The list of FRGs were obtained from the FerrDB database [19]. The database included driver, suppressor and marker genes of ferroptosis. In this study, 140 ferroptosis genes included above types were obtain from the FRGs dataset. Supplemental Table 1 provided the list of FRGs.

### Data collection and differential expression analysis of FRGs

LC cohorts of the TCGA (https://portal.gdc.cancer.gov/) and GEO (https://www.ncbi.nlm.nih.gov/geo/) were used in the present study. Ethical review and approval by the ethics committee was not necessary as this study adhere strictly to the TCGA and GEO database policies and guidelines of data access.

The data used included mRNA expression profiles, clinical data, and survival information of patients. The count data were used for variance analysis, and the log2-FPKM data were used for model construction. To avoid batch effects between different cohort studies on model construction, log2-FPKM values were adjusted for batch effects using sva package [20]. The mRNA expression profiles in TCGA LC cohort matched with FRGs, and the differentially expressed FRGs were confirmed using the limma package [21] by comparing the mRNA expression between LC and Para cancer tissues. Heatmap of 140 FRGs expression was visualized by the heatmap package. The threshold was absolute log2-fold change (FC) ≥ 1 and *P*-value < 0.05.

GSE72094 [22] and GSE157011 [23] were utilized as external validation datasets for the prognostic risk score. GSE72094 and GSE157011 contained gene expression data of 442 lung adenocarcinoma (LUAD) and 484 lung squamous carcinoma (LUSC) samples, and prognostic information of the corresponding patients, respectively. Deseq2 package analyzed the differentially expressed genes in GSE72094 and GSE157011 datasets [24].

### Establishment and verification of FRGs prognostic model

In the light of the differential FRGs, a univariate Cox regression model was applied to screen FRGs related with OS in LC. The genes were screened by the least absolute shrinkage and selection operator (LASSO) regression and intersected with univariate Cox regression models, and multivariate Cox regression analysis was carried on intersected genes to build prognostic risk scores of FRGs. Based on a multivariate Cox regression model and genetic coefficients, the prognostic risk score associated with ferroptosis (the linear part of the Cox regression model) was calculated for each patient in the TCGA cohort, and patients were classified into high- and low-risk groups. Kaplan–Meier (K-M) survival curves was utilized to analyze the differences in OS. Further, the predictive ability of FRGs prognostic risk scores was assessed by receiver operating characteristic (ROC) curves [25].

### Construction and evaluation of forecast nomogram

Univariate and multifactorial Cox regression were applied to analyze the relationship between risk scores, clinical characteristics and prognostic risk to determine whether prognostic characteristics predicted OS independently of other traditional clinical characteristics. Traditional clinical characteristics include age, gender, tumor type, presence of new tumors after initial treatment and TNM stage. These independently clinical features were used to build a forecast nomogram. The predicted nomogram and corresponding calibration curves were constructed by the rms package [26]. Furthermore, we assessed clinical decision-making of the value of nomogram through decision curve analysis (DCA) [27, 28].

### Analysis of immune infiltration

CIBERSORT algorithm is commonly used to define the cellular composition of complex tissues from gene expression profiles [29]. In TCGA LC cohort, the proportion of immune cell was analyzed by CIBERSORT, and immune cell infiltration was assessed using the TIMER method [30], and the differences of immune cell infiltration between high- and low-risk groups were analyzed.

### Clinical tissues and cells

A total of 30 pairs of LC and paired para cancer tissues were collected at the Third Affiliated Hospital of Kunming Medical University. Para cancer tissues were obtained at a distance of at least 3 cm from the LC tissue. All patients did not receive radiotherapy or chemotherapy. Patient information was shown in Table 1. The study was approved by the Ethics Committee of the Third Affiliated Hospital of Kunming Medical University, and all patients have signed an informed consent form.

**Table 1:** Clinical information of 30 LC samples

Characteristics	Number of cases
Gender	
Male	19 (63.3%)
Female	11 (36.7%)
Age(year)	
≥ 60	18 (60.0%)
< 60	12 (40.0%)
Subtype	
LUAD	17 (56.7%)
LUSC	13 (43.3%)
Tumor size (cm)	
≥ 5	6 (20.0%)
< 5	24 (80.0%)
Tumor invasion depth	
T1-2	25 (83.3%)
T3-4	5 (16.7%)
Lymph node metastasis	
N0	18 (60.0%)
N1-2	12 (40.0%)
TNM stage	
I+ II	24 (80.0%)
III + IV	6 (20.0%)
Smoking history	
Yes	13 (43.3%)
No	17 (56.7%)

The cells used in this study included human normal lung epithelial cells (BEAS-2B) and LC cell lines (A549, H1975, HCC827, PC9), which were purchased from the Institute of Biochemistry and Cell Biology, Chinese Academy of Sciences (Shanghai, China). Cells were cultured in DMEM medium (Hyclone, South Logan, UT, USA) containing 10% fetal bovine serum, 100 U/mL penicillin and 100 mg/mL streptomycin at 37 ℃ with 5% CO_2_.

### Immunohistochemistry (IHC) assay [31]

IHC was conducted to observe the proportion of positive area of 8 FRGs in LC and paired para cancer tissues. Briefly, 10% formalin was used to fix tissues of LC and paired para cancer, and 4 µm-thick paraffin sections were prepared. Sections were incubated with 3% H_2_O_2_ at room temperature for 15 min and blocked with 5% normal goat serum. After standing for 20 min, samples were incubated with primary antibodies and biotin-coupled secondary antibodies. Color development was performed with DAB kit (Solarbio, China). Finally, the sections were observed and photographed under a light microscope (CI60; Nikon, Tokyo, Japan). The percentage of positive area for FRGs was calculated using Image J software (National Institutes of Health, USA). ACSL3, CDKN1A, FADS2, HSF1, PANX1, VDAC2 primary antibodies and biotin-coupled secondary antibodies were purchased from Abcam (Cambridge, UK). GLS2 and PHKG2 primary antibodies were purchased from Bioss (Beijing, China). Antibody information was shown in Table 2.

**Table 2:** Antibody information for IHC and Western blotting

Antibody	IHC		Western blotting	
	Cat. No	Dilution ratio	Cat. No	Dilution ratio
Primary antibody				
ACSL3	ab151959	1:200	ab151959	1:1000
CDKN1A	ab102013	1:200	ab172442	1:2000
FADS2	ab262943	1:40	ab232898	1:1000
GLS2	bsm-51645 m	1:25	ab52757	1:50,000
HSF1	ab52757	1:250	ab113509	1:2000
PANX1	ab233479	1:200	ab124969	1:3000
PHKG2	bs-5010R	1:300	ab167424	1:1000
VDAC2	ab126120	1:150	ab155803	1:500
GAPDH	–	–	ab8245	1:1000
Second antibody				
Goat Anti-Rabbit IgG H&L	ab6721	1: 1000	ab6721	1: 2000
Goat Anti-mouse IgG H&L	ab6708	1: 1000	ab6708	1: 3000

### Reverse transcription quantitative real-time polymerase chain reaction (RT-qPCR) assay [32]

Total RNA was extracted from human normal lung epithelial cells and LC cells using TRIzol reagent (TaKaRa, Tokyo, Japan). NanoDrop ND-1000 (Thermo Fisher Scientific, Waltham, MA, USA) was used to determine RNA concentration. Total RNA (500 ng) was reversed transcribed to cDNA through the PrimeScript RT kit (TaKaRa, Tokyo, Japan). Referring to the SYBR Premix Ex Taq kit instructions (TaKaRa, Tokyo, Japan) and using GAPDH as an internal control, PCR was performed by an ABI 7500 real-time fluorescent quantitative PCR instrument system (Applied Biosystems, USA) to detect ACSL3, CDKN1A, FADS2, GLS2, HSF1, PANX1, PHKG2 and VDAC2 mRNA expression. The 2^−ΔΔCt^ method was used to calculate the relative expression level of mRNAs. The primer sequences were displayed in Table 3.

**Table 3:** Primer sequences for RT-qPCR

Name	Forward primer (5ʹ-3ʹ)	Reverse primer (5ʹ-3ʹ)
ACSL3	TGACACAAGGGCGCATATCT	CCAGTCCTTCCCAACAACGA
CDKN1A	AGAATCCATGGTCCAAGGGC	CACCCTGCCCAACCTTAGAG
FADS2	GATGCAACGCACATTTCCAGT	AGTGGCGATGATTCCACCAG
GLS2	CCACTTTCCTCCCCATTCTCTG	TGCATCTCGCTCATGCAGTC
HSF1	TACAGCAGCTCCAGCCTCTA	CACTGAGCTCGGTCTTGTCC
PANX1	CAGCTTTCCCGACGCC	AGAACACGTACTCCGTGGC
PHKG2	CCATGGATGAAACCCACCCA	ATCCACTGCTTCCTTATCCAGT
VDAC2	GCGCGTCCAATGTGTATTCC	AGGAAGACAGCTGATGTGAAA
GAPDH	AGATTGTCAGCAATGCCTCCT	CTCTCTCTTCTTCCTCTTGCTGGG

### Western blotting assay [33]

BEAS-2B cells and LC cells were collected, and total protein was extracted using RIPA lysate (Thermo Fisher Scientific, Waltham, MA, USA). BCA kit (Beyotime, Shanghai, China) was used to determine the concentration of total cellular protein. Sodium Dodecyl Sulfate Polyacrylamide Gel Electrophoresis was used to separate protein, and then the protein was transferred to polyvinylidene fluoride (PVDF) membrane. After blocking the PVDF membrane with 5% bovine serum albumin, the PVDF membrane was incubated with diluted primary and biotin-coupled secondary antibodies. Enhanced chemiluminescence reagent (ThermoFisher Scientific, Waltham, MA, USA) was applied for development, and observation and photography were performed by a ChemiDoc XRS + gel imaging system (Bio-Rad, Hercules, CA, USA). GAPDH was used as the internal reference. Analysis of the target protein was performed by Image J software (National Institutes of Health, Bethesda, MD, USA). All antibodies above were bought from Abcam (Cambridge, UK). Antibody information was shown in Table 2.

### Statistical analysis

Statistical analysis was performed using R software with version 4.1.1. Gene expression of LC tissues was compared with normal tissues using Student’s *t*-test. Immune cells in the high-risk and low-risk groups were compared using the Mann–Whitney test, and *P* values were adjusted with Benjamini-Hochberg. Log-rank statistical tests were utilized to analyze the differences of OS. *P* < 0.05 was considered statistically significant.

## Results

**Fig. 1:**
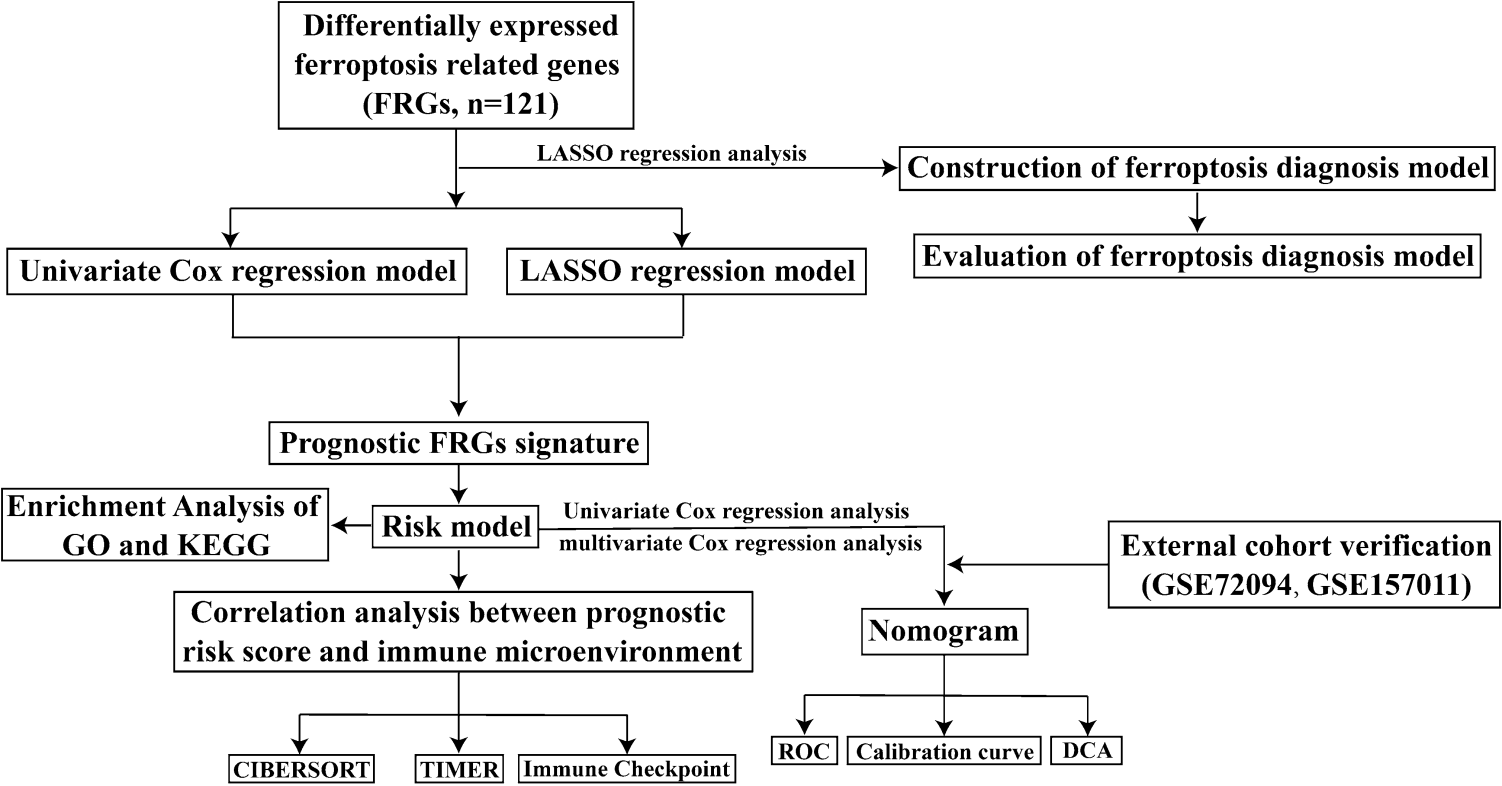
Flowchart of data collection and bioinformatics analysis

The flowchart of this study is shown in Fig. 1. We analyzed the differential genes associated with ferroptosis in the TCGA LC cohort, constructed and evaluated prognostic risk scores, and constructed clinical prognostic and diagnostic models associated with ferroptosis. Meanwhile, the differences of immune infiltration between high-risk and low-risk groups were analyzed to study the relationship between prognostic risk scores and immune checkpoints. In addition, the results of the previous analysis were further validated by the detection of the expression of eight FRGs associated with lung cancer prognosis in clinical samples tissues and cells.

### Differential expression analysis of FRGs in TCGA cohort

**Fig. 2:**
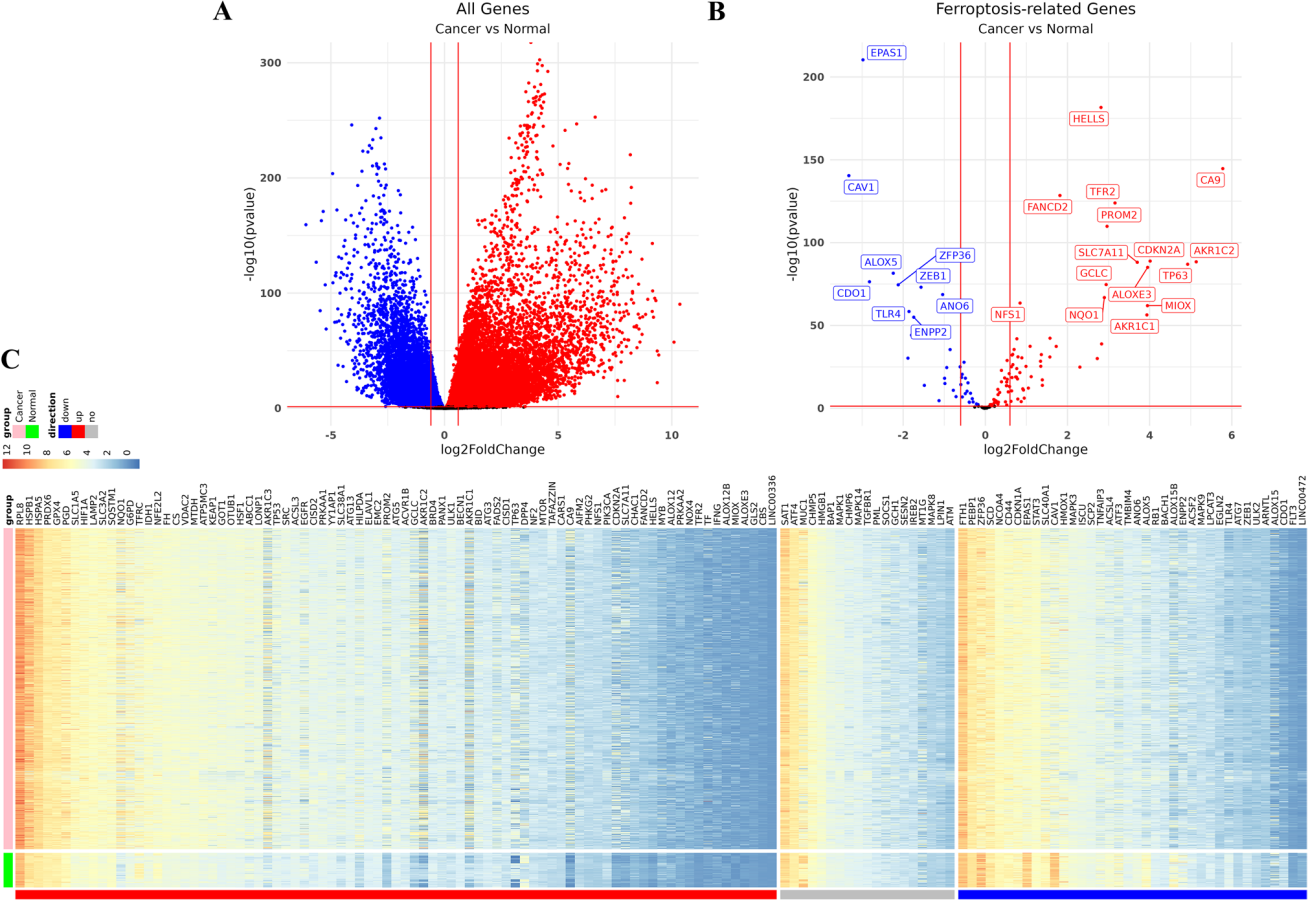
Differential expression analysis of FRGs in TCGA cohort. **A** Volcano plot showed DEGs in TCGA lung cancer cohort. Red means high-expressed DEGs, and blue means low-expressed DEGs. **B** Volcano plot exhibited differential FRGs in DEGs. **C** Heatmap for the expression of 140 FRGs in LC samples and normal samples

The results of differential expression analysis exhibited that there were 15,633 differentially expressed genes (DEGs) between tumor samples and normal samples, including 11,832 expressed up-regulated genes and 3801 expressed down-regulated genes (Fig. 2A). Among these differential genes, 121 (86.43%) FRGs were significantly different, including 83 (59.29%) up-regulated FRGs and 38 (27.14%) FRGs with down-regulated expression (Fig. 2B; Supplemental Table 1). The expression of 140 FRGs in each tissue (log2-FPKM) was shown in Fig. 2C and Supplemental Table 2.

### Construction of prognostic risk scores associated with ferroptosis

**Fig. 3:**
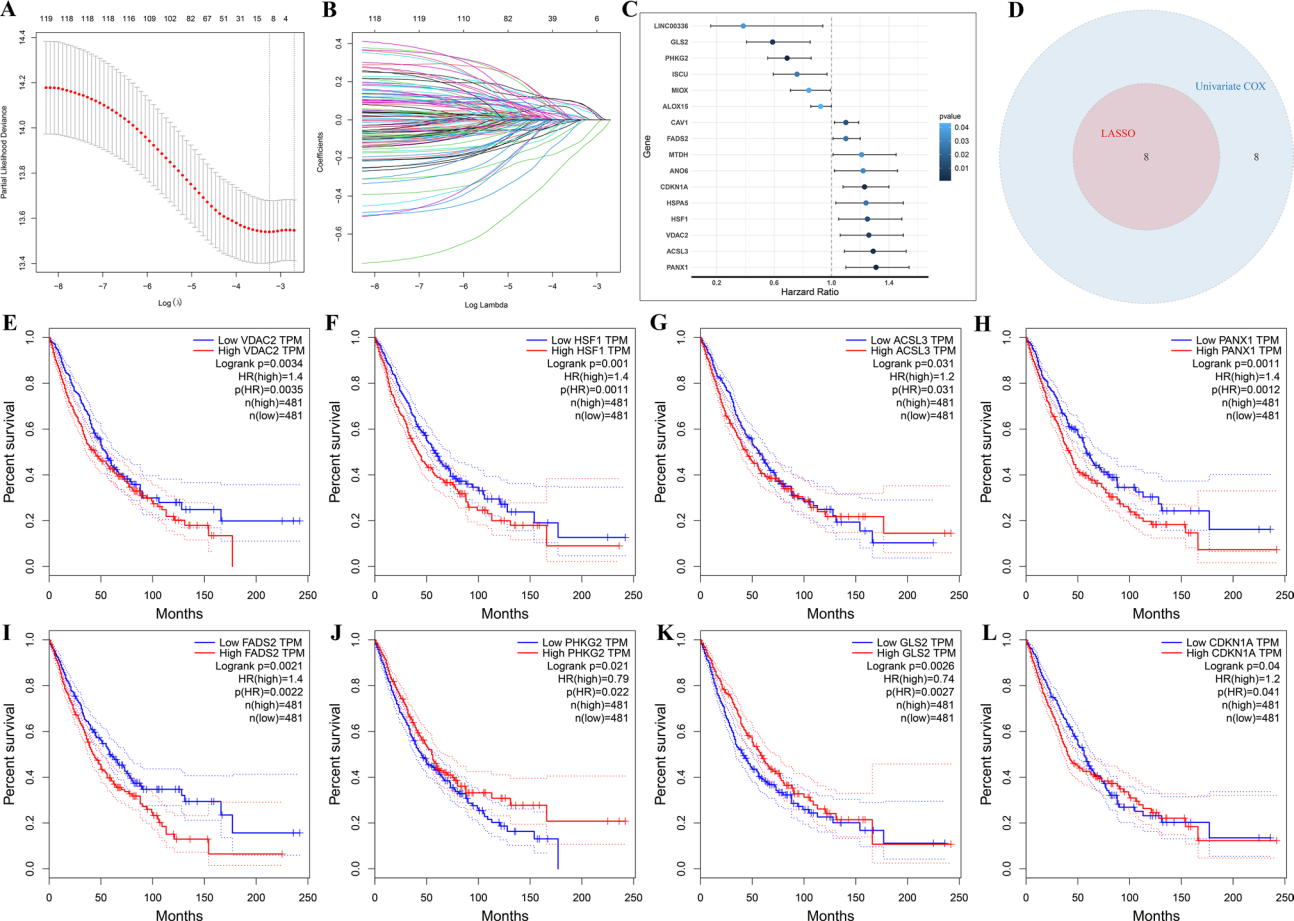
Construction of prognostic risk scores associated with ferroptosis. **A** Screening for the best LASSO model parameter λ. **B** Changes in the number of variables in the LASSO model. **C** Forest plot for genes related to prognosis. **D** Intersection between genes in LASSO model univariate Cox model (HR test *P* < 0.05). **E**–**L** The K-M survival curve of 8-FRGs in the GEPIA database

LASSO regression analysis was used to build a Cox regression model with penalty terms between the expression of 121 FRGs and patient prognosis (Fig. 3A, B). The results confirmed the optimum λ was 0.038624 and there was a total of 8 FRGs with non-zero coefficients in the model with this parameter (Supplemental Table 3). Then, univariate Cox regression model was used to examine whether it was related with the prognostic risk of the patients. Univariate Cox regression models identified a total of 16 genes whose expression could be used to predict prognostic risk (Fig. 3C; Supplemental Table 4). Finally, we took the intersection of the genes obtained from the LASSO regression and univariate Cox regression model, and obtained 8-FRGs significantly associated with LC prognosis, including VDAC2, HSF1, ACSL3, PANX1, FADS2, PHKG2, GLS2 and CDKN1A (Fig. 3D). The multivariate Cox regression analysis was performed on 8 FRGs to construct a prognostic risk score of FRGs. In the light of multivariate Cox regression model and 8-FRGs coefficients (Supplemental Table 5), patients were classified into high- and low-risk groups.

We further analyzed by HPA database [34], and found that the positive rates of VDAC2, HSF1, ACSL3, PANX1, GLS2, and CDKN1A proteins were higher in LC tissues than in normal lung tissues, while the opposite was true for FADS2 protein (supplemental Fig. 1). Unfortunately, no PHKG2 protein information was found in the HPA database.

In addition, the correlation between 8-FRGs and OS in LC was analyzed through the GEPIA database [35]. The results showed that high expression of VDAC2, HSF1, ACSL3, PANX1, FADS2, and CDKN1A predicted a poor prognosis, while high expression of PHKG2 and GLS2 had a good prognosis (Fig. 3E-L).

### Evaluating prognostic risk scores associated with ferroptosis

**Fig. 4:**
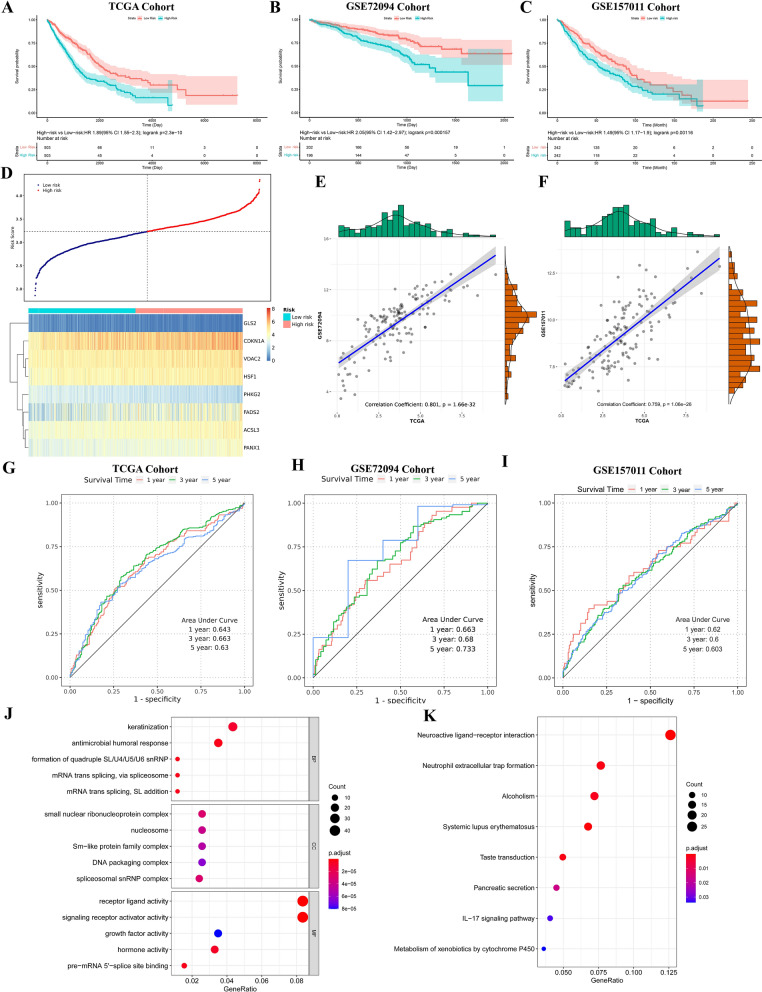
Evaluation of prognostic risk score associated with ferroptosis. **A**–**C** K-M curve indicated OS in high- and low-risk group of (**A**) TCGA, **B** GSE72094, and (**C**) GSE157011 cohorts. **D** The distribution of patient’s risk scores under the expression characteristics of 8-FRGs in the LC cohort from TCGA. **E**, **F** Correlation of 140 FRGs expression in LC cohort of TCGA between (**E**) GSE72094 and (**F**) GSE157011. **G**, **I** The time-dependent ROC curve analysis used to measure OS prediction performance in the (**G**) TCGA, **H** GSE72094, and (**I**) GSE157011 cohorts. **J** GO and (**K**) KEGG pathway enrichment analysis of DEGs in high- and low-risk groups

K-M curves were used to analyze the survival differences. The results demonstrated that, compared with the low-risk group, OS was statistically dwindled in the high-risk group (Fig. 4A; *P* = 2.3e-10, HR = 1.89, 95% CI 1.55–2.3). Figure 4D showed the distribution of risk scores for 8-FRGs expression levels. The above results suggested a significant association between ferroptosis-related prognostic risk score and the survival of patients with LC. Further, ROC curves assessed the predictive ability of ferroptosis-related prognostic risk scores for 1-year, 3-year, and 5-year in patients with LC.

The results indicated that the time-dependent area under the curve (AUC) for 1, 3, and 5 years was 0.643, 0.663, and 0.63, respectively (Fig. 4G). This suggested that the prognostic model had high specificity and sensitivity for predicting OS.

The predictive performance of the prognostic model was then validated through the external cohort GSE72094 and GSE157011. Correlation analysis exhibited that the expression of 121 FRGs in TCGA correlated well with the GSE72094 (Fig. 4E; *P* = 1.66e-32) and GSE157011 (Fig. 4F; *P* = 1.06e-26) cohorts, including 8-FRGs. K-M curves showed that, compared with the low-risk group, OS was dramatically decline in the high-risk group of GSE72094 (Fig. 4B; *P* = 1.57e-4, HR = 2.05, 95% CI: 1.42–2.97) and GSE157011 (Fig. 4C; *P* = 1.16e-3, HR = 1.49, 95% CI 1.17–1.9) cohorts, which was consistent with the TCGA cohort. In GSE72094 cohort, the ROC curves displayed that the AUCs for 1, 3, and 5 years were 0.663, 0.68, and 0.733, respectively (Fig. 4H). In GSE157011 cohort, the ROC curves showed AUCs for 1, 3, and 5 years were 0.62, 0.6, and 0.603, respectively (Fig. 4I).

Further, we performed DEGs analysis for high- and low-risk groups, and GO and KEGG pathway enrichment for DEGs. GO enrichment results indicated that the main enriched terms involved DEGs including “keratinization”, “small nuclear ribonucleoprotein complex” and “receptor ligand activity” (Fig. 4J). The main pathways involved with DEGs including “neuroactive ligand-receptor interaction”, “neutrophil extracellular trap formation”, and “alcoholism” (Fig. 4K).

### TIME comparison between high-risk and low-risk groups

**Fig. 5:**
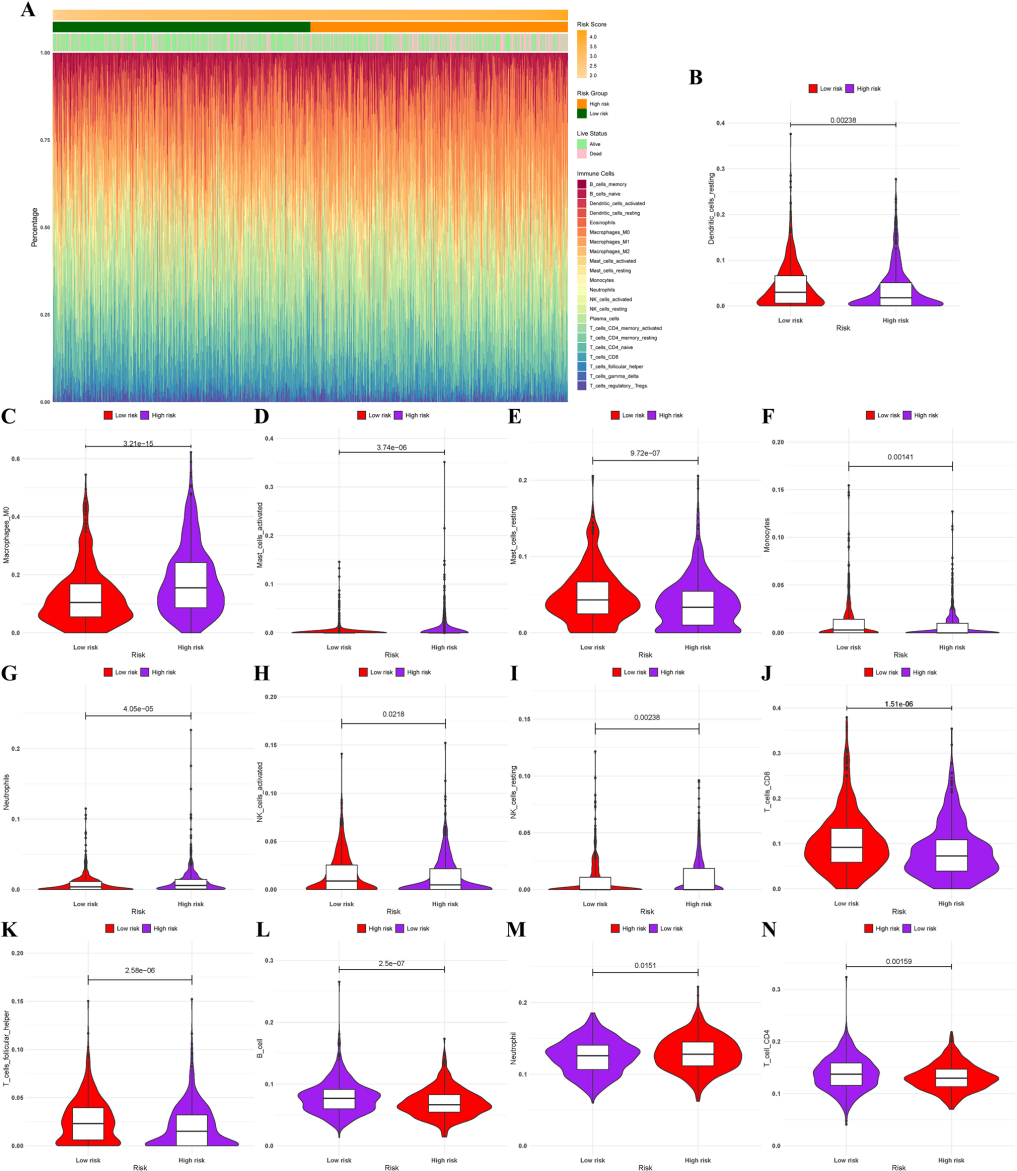
Correlation analysis of immune cells with high- and low-risk groups. **A** Heatmap showed immune infiltration difference of 22 immune cells between high-risk and low-risk groups. **B**–**N** Violin plots displayed the fraction of different immune cells

According to the CIBERSORT algorithm [29], we analysed the immune cell infiltration in 1004 LC patients of TCGA and the differences between high- and low-risk groups (differentiated by prognostic risk score for ferroptosis). Figure 5A displayed the differenced in immune infiltration of 22 immune cells between the high- and low-risk group. There was significant difference in immune infiltration between the two groups (*P*.adj < 0.05), including Dendritic_cells_resting, Macrophages_M0, Mast_cells_resting, Mast_cells_activated, Natural killer (NK)_cells_activated, Monocytes, NK_cells_resting, Neutrophils, T_cells_CD8 and T_cells_follicular_helper. Among them, Neutrophils, Mast_cells_activated, Macrophages_M0 and NK_cells_resting ratios were significantly upregulated in the high-risk group compared with low-risk group; Dendritic_cells_resting, Mast_cells_resting, Monocytes, NK_cells_activated, T_cells_CD8 and T_cells_follicular_helper ratios were significantly down-regulated in the high-risk group (Fig. 5B–K). Moreover, we assessed immune cell infiltration in tumor samples by the TIMER algorithm [30]. The results verified that, compared with the low-risk group, immune infiltration of B cells and CD4+ T cells was lessened in the high-risk group, while Neutrophils were significantly increased (Fig. 5L–N). Supplemental Fig. 2 showed the correlation of 8-FRGs obtained from TIMER database analysis with tumor purity and 6 immune cell infiltrates in LUAD and LUSC, respectively.

**Fig. 6:**
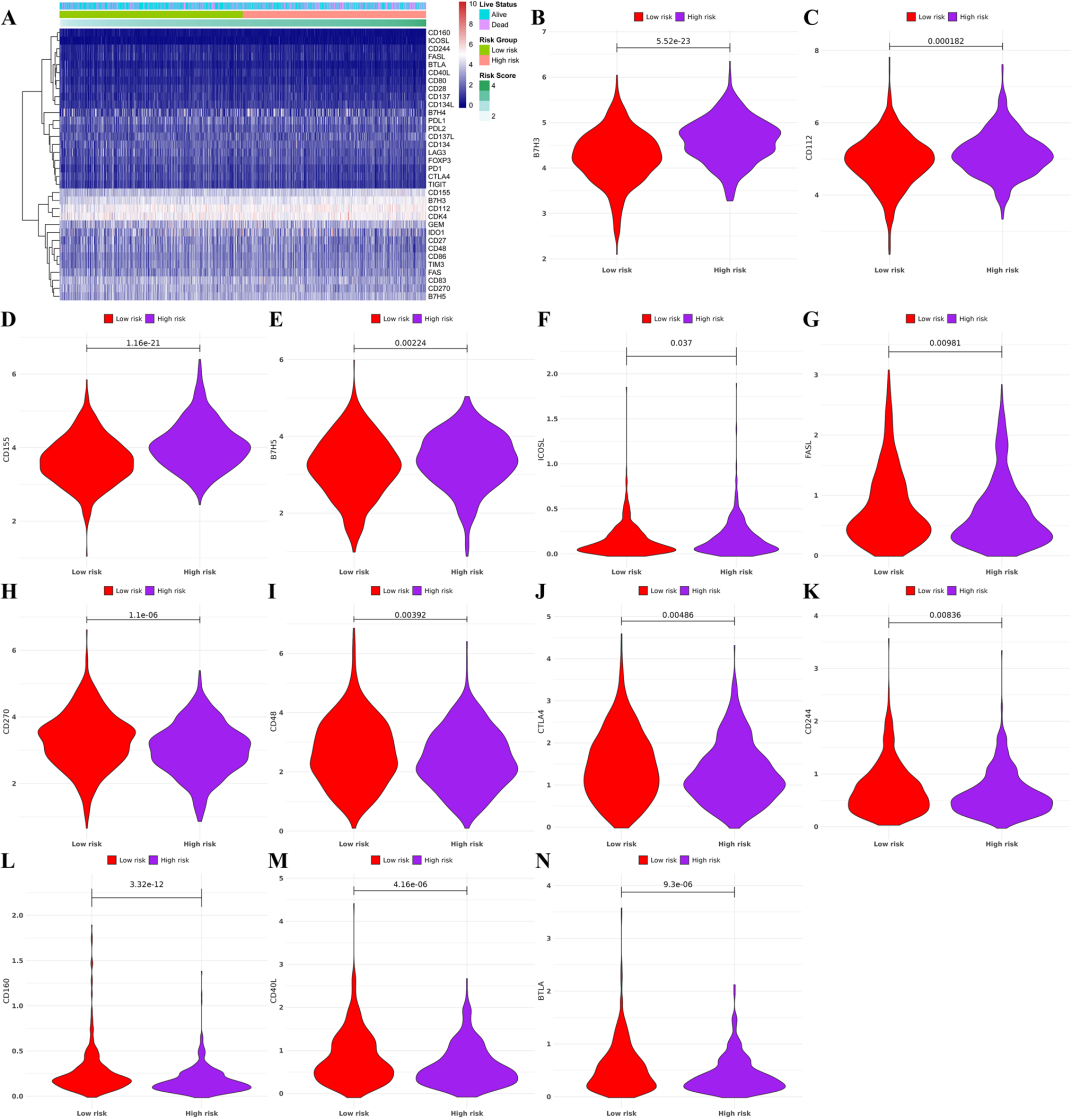
Correlation analysis of immune checkpoints with high- and low-risk groups. **A** Heatmap indicated the differential expression of immune checkpoints. **B**–**N** Violin plots displayed the immune checkpoints with differential expression

We confirmed the relationship between risk score and the expression of immune checkpoint. As shown in Fig. 6A, the results revealed that, compared with the low-risk group, the expression of B7H3, CD112, CD155, B7H5, and ICOSL were significantly upregulated in high-risk group (Fig. 6B–F), and FASL, CD270, CD48, CTLA4, CD244, CD160, CD40L, and BTLA were significantly decreased in the high-risk group (Fig. 6G–N).

### Construction and validation of predictive nomogram for LC from the TCGA cohort

**Fig. 7:**
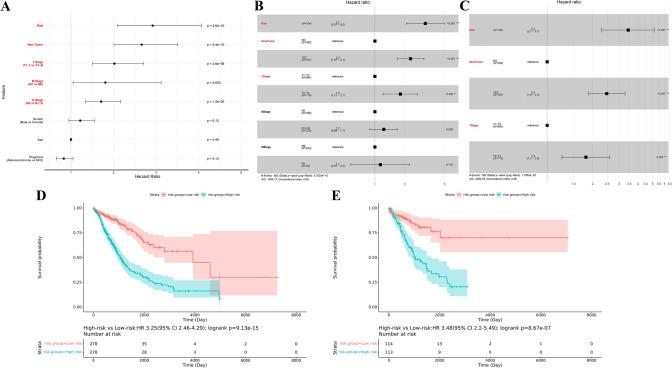
Construction and validation of prognostic model of LC cohort from the TCGA. **A**–**C** Cox regression demonstrated that T stage, NewTumor, and prognostic characteristics were independently prognostic forecasters. **D** and **E** Survival differences between the high-risk group and low-risk group in (**D**) training cohort and (**E**) validation cohort from the TCGA

To evaluate whether the ability of prognostic models to predict OS is independent of other traditional clinical features, we divided the TCGA cohort into training and validation cohort (with the ratio of 7:3; 704 cases in training cohort and 302 cases in validation cohort). The results revealed that the prognostic risk score associated with ferroptosis, TNM stage, and new tumors after initial treatment were prognostic risk factors (Fig. 7A; Supplemental Table 6). As displayed in Fig. 7B, the coefficients of N stage and M stage were not significant in the multivariate Cox model, indicating that these two prognostic risk factors were not independent and the prognostic predictive information they contained could be covered by the other factors in the model. After removing these two factors, a new prognostic risk model was obtained. As shown in Fig. 7C, all prognostic risk factors in this model were independent and the coefficients of each factor were statistically significant. Therefore, this model was used as the final constructed prognostic risk model for LC. The K-M curve results verified that the prognostic risk model could be used to predict the prognostic risk of LC, with dramatically reduce OS in the high-risk group (Fig. 7D; *P* = 9.13e-15, HR = 3.25, 95 CI 2.46–4.29). Prognostic models incorporating clinical information from patients had better predictive performance compared with the prognostic risk score for ferroptosis.

**Fig. 8:**
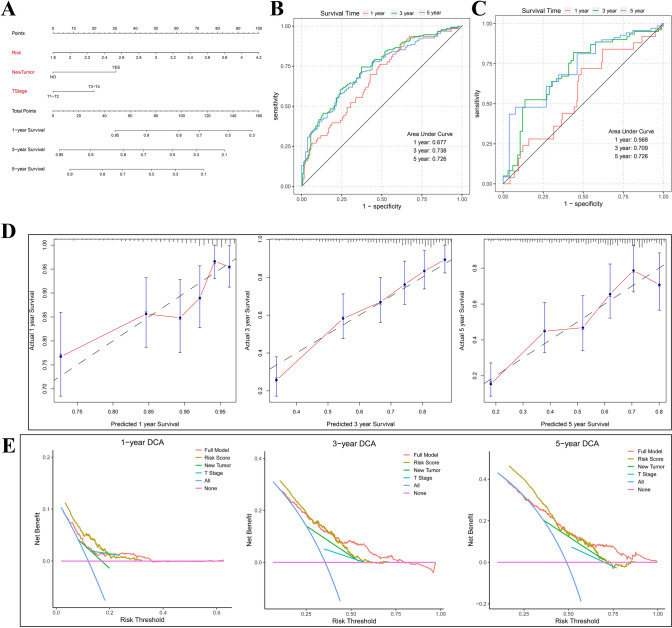
Construction and validation of predictive nomogram of LC cohort from the TCGA. **A** A nomogram for predicting OS of LC at 1, 3, and 5 years. **B**, **C** ROC curve was used to emphasize the predictive performance of nomogram in (**B**) training and (**C**) validation cohort at 1, 3, and 5 years. **D** Calibration curves of the nomogram for OS prediction. **E** DCA curve was used to determine that the nomogram can provide the best clinical decision-making benefits

Based on these three independent predictors, we constructed a predictive nomogram to quantify the predicted outcome of individual 1-, 3-, and 5-year survival probabilities (Fig. 8A). The ROC curves showed the AUCs of OS in the Nomogram for the 1, 3, and 5-year were 0.677, 0.738, and 726, respectively (Fig. 8B), which superior to the individual independent predictors. The calibration curve of the nomogram indicated a good agreement between the predicted OS rate and the actual observed values at 1, 3, and 5 years (Fig. 8D). Subsequently, we performed a DCA to determine the value of the nomogram in clinical decision-making. The DCA curve demonstrated that, compared with New Tumor and T Stage, nomogram could obtain more net beneficial at 1, 3, and 5 years for patients (Fig. 8E). The clinical prognostic risk model of validation cohort also had good predictive performances (Figs. 7E and 8C). The results of validation cohort and training cohort were close, indicating that the model did not overfit the training cohort and has good generalization ability.

### Establishment and validation of TCGA cohort LC samples diagnostic model

**Fig. 9:**
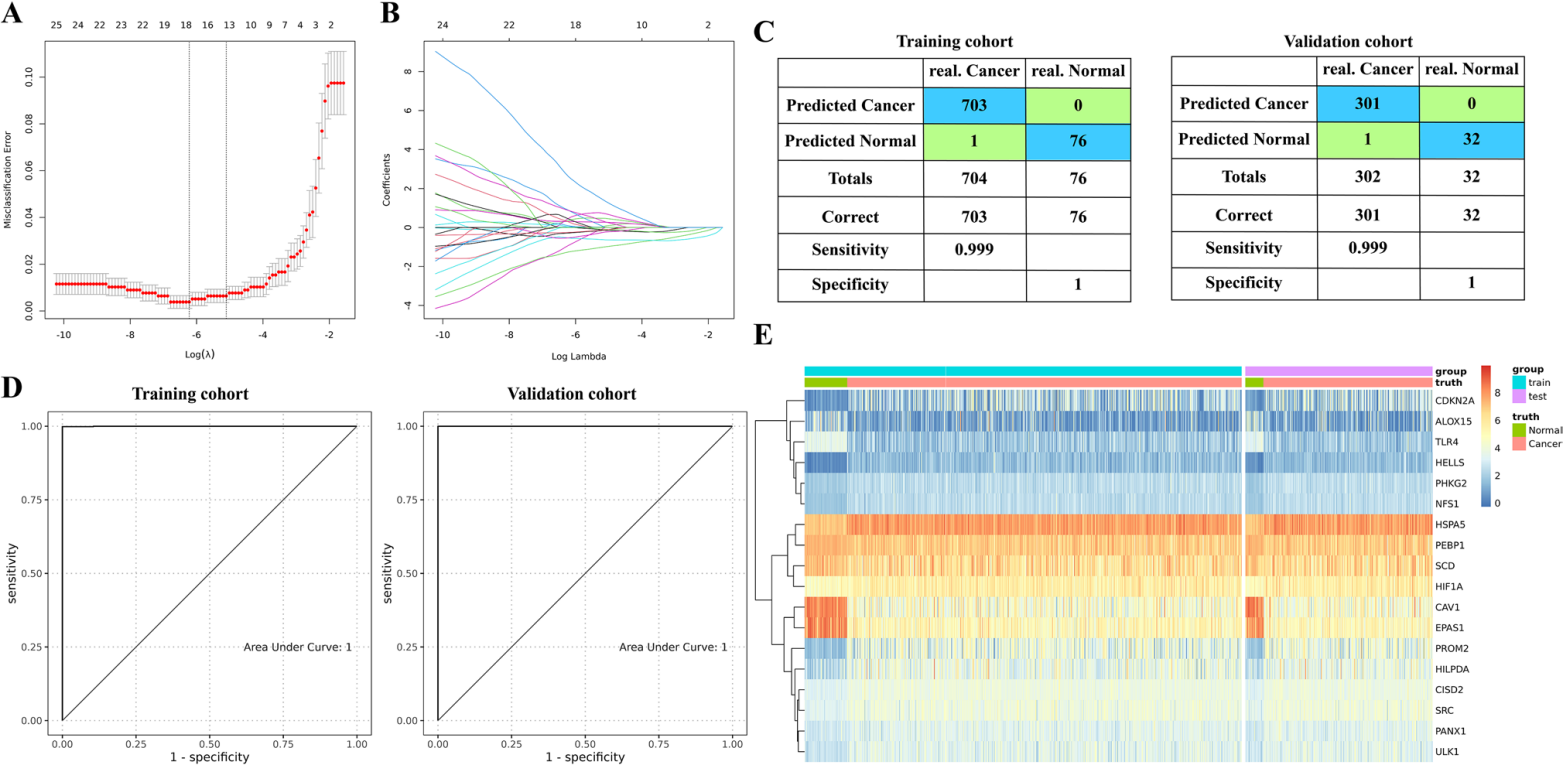
Establishment and validation of diagnostic models for LC cohort from TCGA. **A**, **B** LASSO regression analysis was used to confirm FRGs closely associated with the LC diagnosis. **C** The confusion matrix indicated the predicted classification with diagnostic model and true classification of the sample. **D** The ROC curve is used to reveal the predictive performance of the diagnostic model. **E** Heatmaps of 18 FRGs expression in the diagnostic model of the training and the validation cohort

LASSO regression analysis was used to construct a LC diagnosis model based on 121 FRGs (Fig. 2B). Firstly, the normal tissue samples were divided into training cohort and validation cohort (with ratio of 7:3, with 76 cases in training cohort and 32 cases in validation cohort). The normal tissue in training cohort or validation cohort were combined with the LC tissue in training cohort or validation cohort samples to form the training and validation cohort of the diagnostic model. The tenfold cross-validation showed the optimal λ was 0.001985151 (Fig. 9A-B). In the model with this parameter, there were 18 genes with non-zero coefficients. Applying the diagnostic model, the training cohort had 99.9% sensitivity and 100% specificity; the validation cohort had 99.9% sensitivity and 100% specificity (Fig. 9C). The AUC of both training cohort and validation cohort was 1 (Fig. 9D). As shown in Fig. 9E, the unsupervised hierarchical clustering of the 18 FGRs in training and validation cohort indicated good ability to distinguish LC from normal samples.

### Expression validation of 8 FRGs

**Fig. 10:**
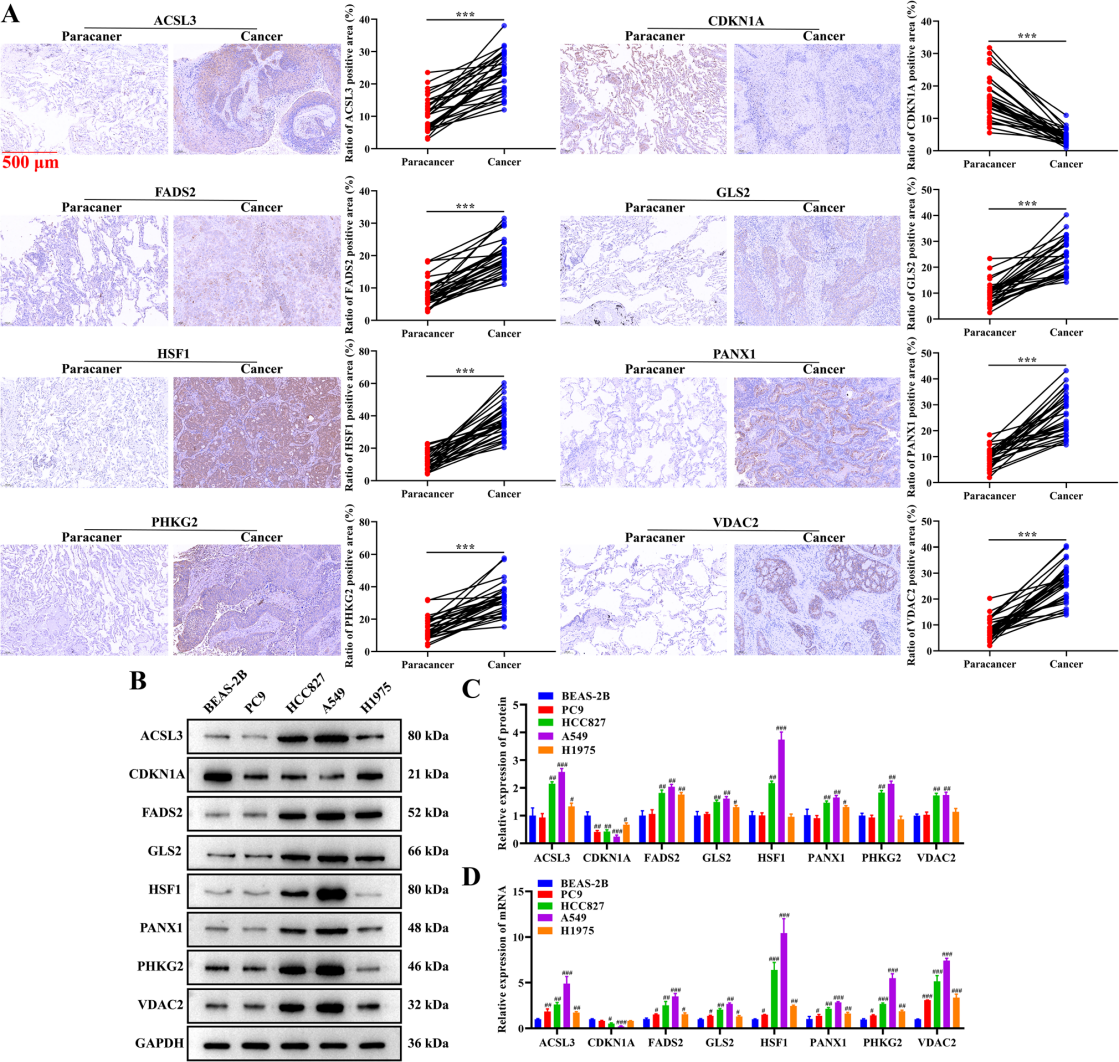
Validation of 8 FRGs expression in clinical samples and cells. **A** Proportion of positive area of 8 FRGs in LC and paired paracancer tissues detected by IHC assay. Scale bar: 100 μm. **B**, **C** Western blotting assay was performed to verify the expression of 8 FRGs proteins in human normal lung epithelial cells (BEAS-2B) and LC cells (PC9, HCC827, A549, H1975). **D** The expression of ACSL3, CDKN1A, FADS2, GLS2, HSF1, PANX1, PHKG2, and VDAC2 mRNA in BEAS-2B cells and LC cells was analyzed by RT-qPCR assay. In all cases, Values are mean ± SD (n = 30 for each group in IHC, n = 3 for each group in Western blotting and RT-qPCR; Compared with Paracancer group, ^***^P < 0.001; Compared with BEAS-2B group, ^#^*P* < 0.05, ^##^*P* < 0.01, ^###^*P* < 0.001)

Further, we examined the expression levels of 8 FRGs associated with the prognosis of LC (Fig. 2D) at the clinical and cellular levels to validate our analysis results. IHC results revealed that the proportion of positive area for ACSL3, FADS2, GLS2, HSF1, PANX1, PHKG2, and VDAC2 were all significantly higher in LC tissues than in paired paracancer tissues, and the proportion of positive area for CDKN1A was lower in LC than that in the paracancer tissues (Fig. 10A). These results were consistent with the finding of differential expression analysis (Fig. 2). In addition, we analyzed the correlation between the expression of 8 FRGs and clinical information of LC samples. The results indicated that ACSL3 and HSF1 were significantly correlated with age, CDKN1A and PANX1 were notably associated with TNM stage. FADS2, PHKG2, and VDAC2 were significantly related to tumor invasion depth. GLS2 was statistically correlated with tumor size and tumor invasion depth (Supplemental Table 7–14). Similarly, Western blotting assay found that ACSL3, FADS2, GLS2, HSF1, PANX1, PHKG2, and VDAC2 proteins were significantly overexpressed in HCC827 and A549 cells, while CDKN1A protein generated an opposite pattern (Fig. 10B and C). RT-qPCR displayed that ACSL3, FADS2, GLS2, HSF1, PANX1, PHKG2, and VDAC2 mRNAs were high expression in all LC cells compared with BEAS-2B cells, while CDKN1A mRNA expression was decreased (Fig. 10D). These results confirmed the finding of differential expression analysis in this study.

## Discussion

LC incidence and mortality rates are increasing year by year, posing a significant health burden to society. Ferroptosis is a novel programmed cell death closely associated with excess iron loading and may have novel molecular mechanisms in tumor immunity and tumor suppression [7]. As previously described that unfavorable prognosis in LC patients was significantly correlated with iron dysregulation [36]. Epidemiological and clinical studies also revealed that iron acts as an essential function in the evolution of LC [37]. Immune system is a dynamic and complex network, and tumor progression and its reaction to treatment are closely monitored by the immune system [38]. Previous studies have shown that cancer cells undergoing ferroptosis could release oxidized lipid that modulate antitumor immunity [10]. Therefore, it is a necessary to identify the key FRGs biomarkers affecting the prognosis of LC, which is of great importance for early diagnosis and improvement of clinical outcome for LC.

In this study, we identified 8-FRGs, and the prognostic model constructed based on 8-FRGs could independently predict the prognosis of LC patients and had good predictive performance. The corresponding nomograms also help clinicians improve clinical decision-making and formulation of treatment plans. In addition, the diagnostic model based on 18-FRGs has high specificity and sensitivity for early diagnosis of LC. In the immune infiltration analysis, we found that the prognostic model had higher proportions of Macrophages_M0, Mast_cells_activated, Neutrophils and NK_cells_resting in the high-risk group than the low-risk group, which indicated the correlation between ferroptosis and TIME, and suggested that the poor prognosis of high-risk groups may be related to strong immunosuppressive effects. Furthermore, we found that immune checkpoints B7H3, CD112, CD155, B7H5, and ICOSL in the high-risk group were increased. These differences promoted the progression of LC and led to poor prognosis of LC.

The 8 FRGs (ACSL3, FADS2, GLS2, HSF1, PANX1, PHKG2, VDAC2, and CDKN1A) that we selected to be associated with the diagnosis and prognosis of lung cancer have been shown to play important roles in cancer and tumor immunity. Acyl‐CoA synthetase long‐chain family member 3 (ACSL3) is a member of the long-chain acyl-COA synthetase family and a lipid-metabolizing enzyme that converts free fatty acids to fatty acid-CoA. The expression of ACSL3 increased in prostate cancer cells, which promoted the growth of CRPC by promoting the synthesis of dehydroepiandrosterone and preventing the catabolism of active androgen [39]. ACSL3 facilitated growth of LC cell, and was exceptionally high expression in LC tissues [40]. ACSL3-mediated fatty acid oxidation was essential for lung carcinogenesis with KRAS mutant [41], and numerous articles have shown that ACSL3 is a key gene in the prognostic model of LUAD [42]. Knockdown of ACSL3 impeded pancreatic ductal carcinoma progression, which regulated fibrotic and ratio of immune cells in TIME [43]. Fatty acid desaturase 2 (FADS2) acted as a desaturating agent mainly by introducing a double bond at the δ6 position of the fatty acid chain, which was the first rate-limiting enzyme for the conversion of upstream fatty acids to PUFA. As previously described, FADS2 was higher expression in LC tissues than in paraneoplastic tissues [44], and knockdown of FADS2 led to a remarkable increase in iron and lipid ROS in LC cells, and eventually LC cells underwent ferroptosis [45]. Glutaminase 2 (GLS2) was a highly mobile and multiple positioning protein that transfer to both the mitochondria and the nucleus, and nuclear translocation of GLS2 was associated with proliferation inhibition and cell differentiation in LC [46]. Dias et al. [47] reported that GLS2 promoted breast cancer development by promoting the proliferation and metastasis of breast cancer cells. What’s more, GLS2 could be used as a therapeutic target of ferroptosis in cardiomyocytes [48]. Heat shock factor 1 (HSF1) as a major regulator in protein homeostasis, and it has been demonstrated to be up-regulated in LC cells, and was necessary for brain metastasis in vivo [49]. A clinical study showed that overexpression of HSF1 was a biomarker of unfavorable prognosis in LC [50]. In tumor immunity, HSF1 inhibition triggers loss of NK cell activation ligand MICA/B [51].

Purines from pannexin 1 (PANX1) was a channel-forming glycoprotein found in tumor cells and other cells in TIME, including immune cells, which played an important role in the exchange of information between cells, due to its main function of forming large-pore single-membrane channels that related release of ATP and metabolites [52]. PANX1 has been revealed to promote metastasis in a variety of tumors including hepatocellular carcinoma [53], testicular cell carcinoma [54], and breast cancer [55]. Phosphorylase kinase G2 (PHKG2) can be used as a biomarker for thyroid cancer [56], endometrial cancer [57], renal clear cell carcinoma [58] and colorectal cancer [59]. However, the function of PANX1 in LC is still to be further investigated. Voltage dependent anion channel 2 (VDAC2) acts as a mediator of oxidative stress response and regulates production of ROS, translocation of Bax and release of cytochrome c during ME-344 (a therapeutic isoflavone)-induced mitochondria-mediated apoptosis in LC cells [60]. Mcl-1 was upregulated in NSCLC, and Mcl-1 promoted migration by increasing mitochondrial Ca2^+^ uptake and ROS production through direct interaction with VDAC2 [61]. Cyclin-dependent kinase inhibitor 1A (CDKN1A) encodes a potent cyclin-dependent kinase inhibitor. At the same time, the expression of CDKN1A is tightly regulated by tumor suppressor protein P53, which mediates p53-dependent cell cycle G1 arrest in response to a variety of stress stimuli [62]. The expression of CDKN1A was increased in NSCLC, and knock-down of CDKN1A can significantly promote apoptosis and G1 phase arrest [63].

Compared with previous prognostic models [64, 65], our prognostic model had a larger sample size and was more comprehensive, and we constructed a diagnostic model for the diagnosis of LC. In this study, the diagnostic and prognostic models have excellent predictive performance, and can help clinicians improve clinical decision-making and formulation of treatment plans. Unfortunately, there are still more limitations in this study. For example, we combined the samples of lung squamous cell carcinoma and LUAD in non-small cell lung cancer for analysis, but the fact was that there were some differences in the prognosis of lung adenocarcinoma and lung adenocarcinoma. In subsequent studies, we will conduct further individual analyses of the subtypes of lung squamous cell carcinoma and lung adenocarcinoma in order to obtain a more rigorous diagnostic and prognostic model. In addition, this study is only retrospective and requires prospective studies to corroborate each other's results; functional experiment of 8 FRGs in LC are lacking for validation.

## Conclusion

We established a prognostic model based on 8-FRGs and a diagnostic model based on 18-FRGs for LC. Diagnostic and prognostic models based on these FRGs have superior diagnostic and predictive performance. Moreover, we revealed a correlation between risk scores of prognostic model and immune cell infiltration in the TIME, which provided potential biomarkers for future studies of ferroptosis and TIME in LC.

### Supplementary Information

Supplementary Material 1: Figure 1 Immunohistochemistry from HPA database revealed expression difference of (A) VDAC2, (B) HSF1, (C) ACSL3, (D) PANX1, (E) FADS2, (F) GLS2 and (G) CDKN1A in LC and normal tissues. Figure 2 Correlations of 8-FRGs with tumor purity, B cells, CD8+ T cells, CD4+ T cells, macrophage, neutrophil and dendritic cells in LUAD and LUSC, respectively.

Supplementary Material 2: Table 1 FRGs list and its difference analysis results. Table 2 The expression of 140 FRGs in each tissue. Table 3 Gene coefficients in LASSO models. Table 4 Differentially expressed 121 FRGs were analyzed by univariate Cox regression analysis. Table 5 Gene coefficients in multivariate Cox models. Table 6 Univariate Cox regression analysis of prognostic and clinical features associated with OS in LC patients. Table 7–14. Correlation between ACSL3, CDKN1A, FADS2, GLS2, HSF1, PANX1, PHKG2 and VDAC2 expression and clinical information of LC.

## Data Availability

The datasets supporting the conclusions of this article are included within the article.
