# Supplementary material for: A ferroptosis-related signature predicts the clinical diagnosis and prognosis, and associates with the immune microenvironment of lung cancer
Source: Discov Oncol. 2024 May 14;15:163. doi: 10.1007/s12672-024-01032-x (PMC11093956; doi:10.1007/s12672-024-01032-x)
Supplement: Supplementary file 1 — Supplementary Material 1: Figure 1 Immunohistochemistry from HPA database revealed expression difference of (A) VDAC2, (B) HSF1, (C) ACSL3, (D) PANX1, (E) FADS2, (F) GLS2 and (G) CDKN1A in LC and normal tissues. Figure 2 Correlations of 8-FRGs with tumor purity, B cells, CD8+ T cells, CD4+ T cells, macrophage, neutrophil and dendritic cells in LUAD and LUSC, respectively. [file 12672_2024_1032_MOESM1_ESM.docx]

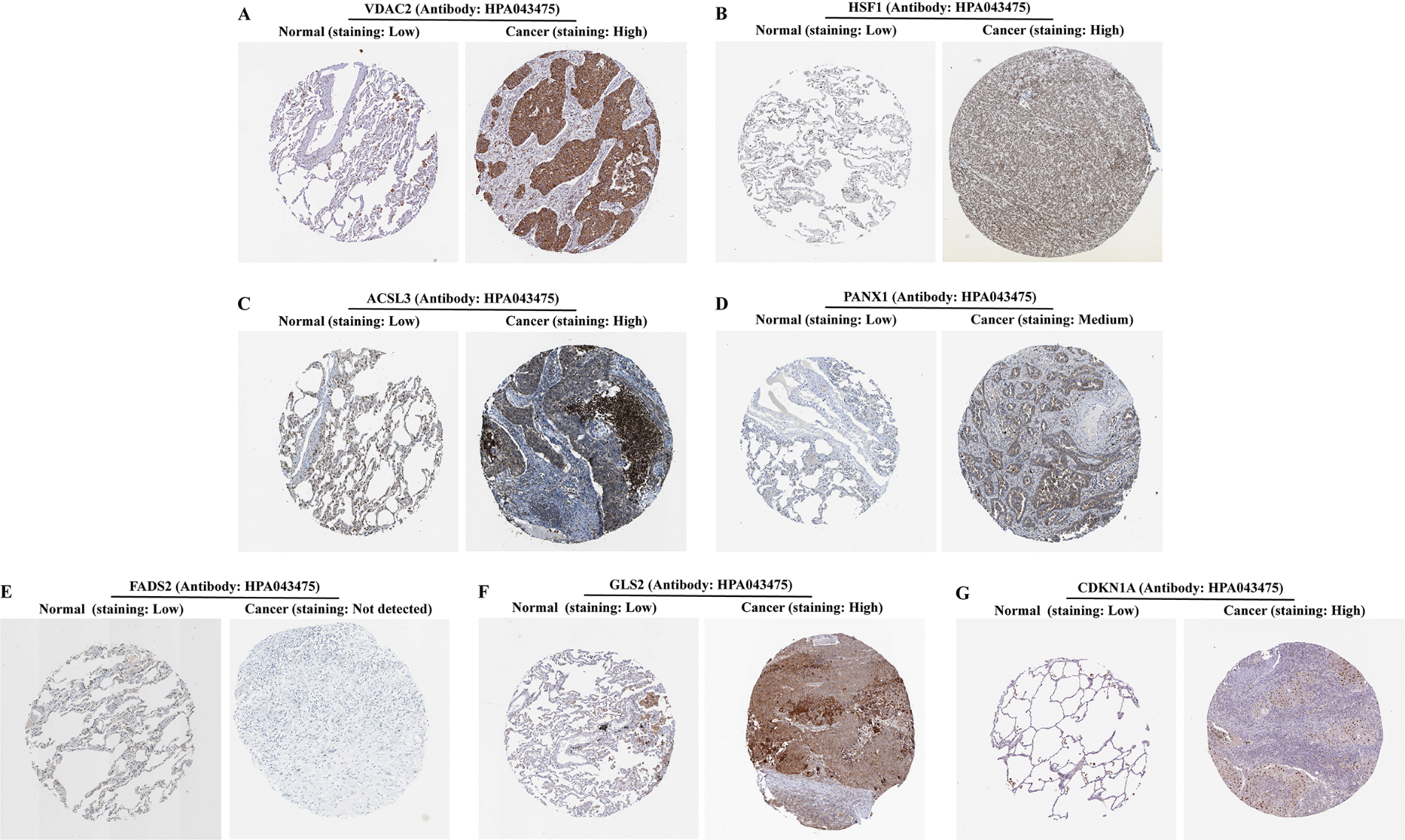


**Supplemental figure 1.** Immunohistochemistry from HPA database revealed expression difference of (A) VDAC2, (B) HSF1, (C) ACSL3, (D) PANX1, (E) FADS2, (F) GLS2 and (G) CDKN1A in lung cancer and normal tissues.


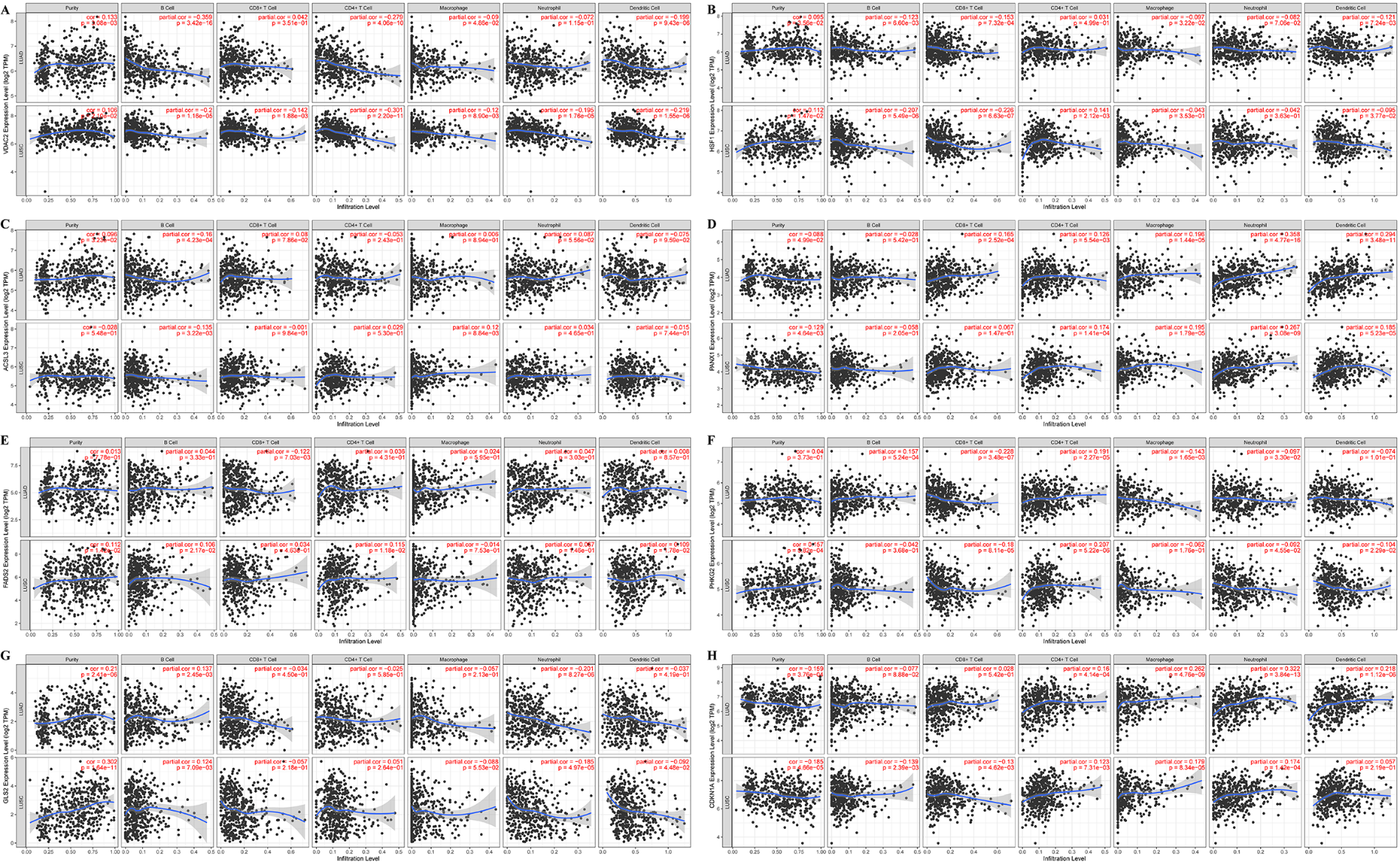


**Supplemental figure 2.** Correlations of 8-FRGs with tumor purity, B cells, CD8^+^ T cells, CD4^+^ T cells, macrophage, neutrophil and dendritic cells in LUAD and LUSC, respectively.
