# Supplementary material for: A ferroptosis-related signature predicts the clinical diagnosis and prognosis, and associates with the immune microenvironment of lung cancer
Source: Discov Oncol. 2024 May 14;15:163. doi: 10.1007/s12672-024-01032-x (PMC11093956; doi:10.1007/s12672-024-01032-x)
Supplement: Supplementary file 2 — Supplementary Material 2: Table 1 FRGs list and its difference analysis results. Table 2 The expression of 140 FRGs in each tissue. Table 3 Gene coefficients in LASSO models. Table 4 Differentially expressed 121 FRGs were analyzed by univariate Cox regression analysis. Table 5 Gene coefficients in multivariate Cox models. Table 6 Univariate Cox regression analysis of prognostic and clinical features associated with OS in LC patients. Table 7–14. Correlation between ACSL3, CDKN1A, FADS2, GLS2, HSF1, PANX1, PHKG2 and VDAC2 expression and clinical information of LC. [file 12672_2024_1032_MOESM2_ESM.docx]

**Supplemental table 1.** FRGs list and its difference analysis results

| GeneID | GeneName | log2FoldChange | p.adj |
| --- | --- | --- | --- |
| ENSG00000001084 | GCLC | 2.941130771 | 2.01E-75 |
| ENSG00000005893 | LAMP2 | 0.261749877 | 3.51E-05 |
| ENSG00000012779 | ALOX5 | -2.241454682 | 2.90E-82 |
| ENSG00000015475 | BID | 0.78315854 | 1.85E-18 |
| ENSG00000026508 | CD44 | -0.392490057 | 0.000183269 |
| ENSG00000042286 | AIFM2 | 0.557732156 | 1.20E-10 |
| ENSG00000044574 | HSPA5 | 0.767485974 | 1.10E-42 |
| ENSG00000050748 | MAPK9 | -0.177543833 | 0.005528084 |
| ENSG00000057663 | ATG5 | 0.219362229 | 0.000366528 |
| ENSG00000062485 | CS | 0.39221282 | 1.01E-16 |
| ENSG00000066044 | ELAVL1 | 0.391926562 | 1.30E-23 |
| ENSG00000068366 | ACSL4 | -1.229576365 | 3.08E-43 |
| ENSG00000072274 | TFRC | 0.624624822 | 6.36E-05 |
| ENSG00000073282 | TP63 | 4.924967722 | 1.09E-87 |
| ENSG00000079999 | KEAP1 | 0.5899074 | 7.26E-14 |
| ENSG00000083290 | ULK2 | -0.857573775 | 3.65E-36 |
| ENSG00000086065 | CHMP5 | 0.034677982 | 0.682385071 |
| ENSG00000086991 | NOX4 | 1.34333372 | 3.86E-29 |
| ENSG00000089220 | PEBP1 | -0.576513687 | 2.33E-19 |
| ENSG00000091483 | FH | 0.666207437 | 2.86E-33 |
| ENSG00000091513 | TF | 2.724738286 | 9.22E-31 |
| ENSG00000099194 | SCD | -0.560655865 | 1.43E-07 |
| ENSG00000100030 | MAPK1 | -0.07889878 | 0.19869661 |
| ENSG00000100253 | MIOX | 3.947028402 | 1.11E-62 |
| ENSG00000100292 | HMOX1 | -0.779425146 | 1.35E-11 |
| ENSG00000100644 | HIF1A | 0.737310385 | 1.26E-19 |
| ENSG00000102125 | TAFAZZIN | 0.534743302 | 3.20E-18 |
| ENSG00000102882 | MAPK3 | -0.615020491 | 9.38E-26 |
| ENSG00000103222 | ABCC1 | 1.349541924 | 4.48E-26 |
| ENSG00000104412 | EMC2 | 0.226318981 | 1.17E-05 |
| ENSG00000105281 | SLC1A5 | 1.089661488 | 3.82E-38 |
| ENSG00000105974 | CAV1 | -3.322550378 | 3.61E-141 |
| ENSG00000106211 | HSPB1 | 1.574095485 | 4.94E-43 |
| ENSG00000106327 | TFR2 | 3.156059676 | 1.15E-124 |
| ENSG00000106799 | TGFBR1 | -0.059168084 | 0.497858606 |
| ENSG00000107159 | CA9 | 5.780263819 | 2.06E-145 |
| ENSG00000107643 | MAPK8 | 0.081938824 | 0.134939749 |
| ENSG00000108839 | ALOX12 | 1.386462762 | 6.87E-18 |
| ENSG00000110218 | PANX1 | 0.512863887 | 2.33E-14 |
| ENSG00000110619 | CARS1 | 0.530055931 | 1.35E-29 |
| ENSG00000111371 | SLC38A1 | 0.989568477 | 4.97E-26 |
| ENSG00000111537 | IFNG | 0.947316558 | 7.22E-07 |
| ENSG00000111684 | LPCAT3 | -0.596041983 | 4.90E-15 |
| ENSG00000112062 | MAPK14 | 0.021438013 | 0.677851691 |
| ENSG00000116016 | EPAS1 | -2.979095759 | 4.46E-211 |
| ENSG00000116044 | NFE2L2 | 0.307596151 | 0.002591739 |
| ENSG00000116171 | SCP2 | -0.360974837 | 8.39E-10 |
| ENSG00000117592 | PRDX6 | 0.224565068 | 0.000259994 |
| ENSG00000118503 | TNFAIP3 | -0.992567983 | 7.50E-19 |
| ENSG00000118513 | MYB | 0.790301702 | 2.64E-06 |
| ENSG00000119969 | HELLS | 2.815493822 | 2.48E-182 |
| ENSG00000120053 | GOT1 | 0.888385922 | 3.47E-31 |
| ENSG00000121879 | PIK3CA | 0.317824655 | 0.000184409 |
| ENSG00000122025 | FLT3 | -0.713000843 | 9.25E-08 |
| ENSG00000122873 | CISD1 | 0.519340837 | 1.34E-16 |
| ENSG00000123983 | ACSL3 | 0.215569484 | 0.000725001 |
| ENSG00000124762 | CDKN1A | -0.939923688 | 2.54E-25 |
| ENSG00000125144 | MT1G | -0.023005066 | 0.916162362 |
| ENSG00000126581 | BECN1 | 0.118973249 | 0.005863227 |
| ENSG00000128016 | ZFP36 | -2.120412963 | 2.90E-75 |
| ENSG00000128272 | ATF4 | 0.095805052 | 0.140787188 |
| ENSG00000128965 | CHAC1 | 1.363391709 | 3.82E-33 |
| ENSG00000129596 | CDO1 | -2.820783048 | 4.16E-77 |
| ENSG00000130066 | SAT1 | -0.167593115 | 0.058789145 |
| ENSG00000130766 | SESN2 | 0.035003835 | 0.666708173 |
| ENSG00000131979 | GCH1 | -0.010548763 | 0.926969811 |
| ENSG00000132356 | PRKAA1 | 0.134488324 | 0.030433196 |
| ENSG00000133794 | ARNTL | -0.215747227 | 0.002199533 |
| ENSG00000134324 | LPIN1 | -0.020168848 | 0.84165156 |
| ENSG00000134824 | FADS2 | 0.48979706 | 0.000125841 |
| ENSG00000135245 | HILPDA | 1.725489719 | 4.28E-38 |
| ENSG00000135423 | GLS2 | 0.911321156 | 1.72E-12 |
| ENSG00000135503 | ACVR1B | 0.522698286 | 1.91E-14 |
| ENSG00000136003 | ISCU | -0.518536901 | 1.60E-28 |
| ENSG00000136381 | IREB2 | 0.058250488 | 0.314557014 |
| ENSG00000136869 | TLR4 | -1.858156236 | 3.74E-59 |
| ENSG00000136960 | ENPP2 | -1.742418071 | 1.03E-55 |
| ENSG00000138413 | IDH1 | 0.755066115 | 1.49E-19 |
| ENSG00000138449 | SLC40A1 | -0.984088863 | 1.05E-15 |
| ENSG00000139687 | RB1 | -0.207493322 | 0.003967799 |
| ENSG00000140464 | PML | 0.103000885 | 0.185450686 |
| ENSG00000141510 | TP53 | 0.639644602 | 3.07E-11 |
| ENSG00000141867 | BRD4 | 0.231590011 | 0.000180659 |
| ENSG00000142657 | PGD | 1.115974935 | 8.12E-20 |
| ENSG00000144554 | FANCD2 | 1.81593353 | 3.40E-129 |
| ENSG00000144848 | ATG3 | 0.150615431 | 0.005471088 |
| ENSG00000145354 | CISD2 | 0.649650927 | 7.61E-33 |
| ENSG00000146648 | EGFR | 1.047090976 | 6.42E-13 |
| ENSG00000147649 | MTDH | 0.348562853 | 2.65E-10 |
| ENSG00000147889 | CDKN2A | 4.011363446 | 1.38E-89 |
| ENSG00000148516 | ZEB1 | -1.565267998 | 8.19E-74 |
| ENSG00000149311 | ATM | -0.038635249 | 0.65892913 |
| ENSG00000151012 | SLC7A11 | 3.698219786 | 7.15E-89 |
| ENSG00000151632 | AKR1C2 | 5.133348009 | 3.74E-89 |
| ENSG00000154518 | ATP5MC3 | 0.764920893 | 1.03E-24 |
| ENSG00000155066 | PROM2 | 2.962590411 | 1.41E-110 |
| ENSG00000155957 | TMBIM4 | -0.487082388 | 1.36E-09 |
| ENSG00000156273 | BACH1 | -0.31805408 | 1.56E-06 |
| ENSG00000156873 | PHKG2 | 0.699447361 | 3.08E-36 |
| ENSG00000160200 | CBS | 1.387917936 | 1.36E-14 |
| ENSG00000160211 | G6PD | 1.570627247 | 1.07E-31 |
| ENSG00000161011 | SQSTM1 | 0.292197836 | 0.000661994 |
| ENSG00000161016 | RPL8 | 0.754683994 | 1.34E-19 |
| ENSG00000161905 | ALOX15 | -1.127627065 | 2.42E-05 |
| ENSG00000162409 | PRKAA2 | 0.927680098 | 9.55E-12 |
| ENSG00000162772 | ATF3 | -1.794935872 | 6.77E-45 |
| ENSG00000163374 | YY1AP1 | 0.304975479 | 3.93E-12 |
| ENSG00000163930 | BAP1 | 0.069054198 | 0.182440852 |
| ENSG00000165637 | VDAC2 | 0.649436273 | 2.65E-19 |
| ENSG00000167107 | ACSF2 | -0.387339049 | 0.000243109 |
| ENSG00000167468 | GPX4 | 0.156532273 | 0.03181639 |
| ENSG00000167770 | OTUB1 | 0.469738698 | 4.58E-19 |
| ENSG00000167996 | FTH1 | -0.297325373 | 0.000145649 |
| ENSG00000168003 | SLC3A2 | 0.840591575 | 2.84E-22 |
| ENSG00000168610 | STAT3 | -0.422493038 | 4.30E-16 |
| ENSG00000175224 | ATG13 | 0.206715308 | 4.84E-06 |
| ENSG00000176108 | CHMP6 | -0.085174588 | 0.183384335 |
| ENSG00000177119 | ANO6 | -1.03896782 | 2.22E-69 |
| ENSG00000177169 | ULK1 | 0.794638627 | 8.91E-32 |
| ENSG00000179148 | ALOXE3 | 3.951013329 | 8.62E-86 |
| ENSG00000179477 | ALOX12B | 2.301192691 | 1.35E-25 |
| ENSG00000179593 | ALOX15B | -1.48462369 | 1.43E-14 |
| ENSG00000181019 | NQO1 | 2.896888655 | 1.39E-67 |
| ENSG00000185122 | HSF1 | 0.635448044 | 8.65E-26 |
| ENSG00000185338 | SOCS1 | 0.223389042 | 0.061109193 |
| ENSG00000185499 | MUC1 | -0.258586494 | 0.16706452 |
| ENSG00000186575 | NF2 | 0.162082043 | 0.009156103 |
| ENSG00000187134 | AKR1C1 | 3.932623311 | 4.33E-57 |
| ENSG00000189403 | HMGB1 | -0.021684828 | 0.74188346 |
| ENSG00000196139 | AKR1C3 | 2.830290734 | 1.38E-39 |
| ENSG00000196365 | LONP1 | 0.580216318 | 1.20E-21 |
| ENSG00000197122 | SRC | 0.670895549 | 4.41E-27 |
| ENSG00000197251 | LINC00336 | 0.872787073 | 9.15E-05 |
| ENSG00000197548 | ATG7 | -0.485366997 | 6.88E-19 |
| ENSG00000197635 | DPP4 | 0.520935098 | 0.010101218 |
| ENSG00000198793 | MTOR | 0.308940516 | 5.55E-09 |
| ENSG00000233237 | LINC00472 | -1.883830661 | 5.47E-31 |
| ENSG00000244005 | NFS1 | 0.845160971 | 2.95E-64 |
| ENSG00000266412 | NCOA4 | -0.562780646 | 2.91E-21 |
| ENSG00000269858 | EGLN2 | -0.444818767 | 4.67E-11 |

**Supplemental table 2.** The expression of 140 FRGs in each tissue.

| GeneID | GeneName | log2FoldChange | p.adj |
| --- | --- | --- | --- |
| ENSG00000001084 | GCLC | 2.941130771 | 2.01E-75 |
| ENSG00000005893 | LAMP2 | 0.261749877 | 3.51E-05 |
| ENSG00000012779 | ALOX5 | -2.241454682 | 2.90E-82 |
| ENSG00000015475 | BID | 0.78315854 | 1.85E-18 |
| ENSG00000026508 | CD44 | -0.392490057 | 0.000183269 |
| ENSG00000042286 | AIFM2 | 0.557732156 | 1.20E-10 |
| ENSG00000044574 | HSPA5 | 0.767485974 | 1.10E-42 |
| ENSG00000050748 | MAPK9 | -0.177543833 | 0.005528084 |
| ENSG00000057663 | ATG5 | 0.219362229 | 0.000366528 |
| ENSG00000062485 | CS | 0.39221282 | 1.01E-16 |
| ENSG00000066044 | ELAVL1 | 0.391926562 | 1.30E-23 |
| ENSG00000068366 | ACSL4 | -1.229576365 | 3.08E-43 |
| ENSG00000072274 | TFRC | 0.624624822 | 6.36E-05 |
| ENSG00000073282 | TP63 | 4.924967722 | 1.09E-87 |
| ENSG00000079999 | KEAP1 | 0.5899074 | 7.26E-14 |
| ENSG00000083290 | ULK2 | -0.857573775 | 3.65E-36 |
| ENSG00000086065 | CHMP5 | 0.034677982 | 0.682385071 |
| ENSG00000086991 | NOX4 | 1.34333372 | 3.86E-29 |
| ENSG00000089220 | PEBP1 | -0.576513687 | 2.33E-19 |
| ENSG00000091483 | FH | 0.666207437 | 2.86E-33 |
| ENSG00000091513 | TF | 2.724738286 | 9.22E-31 |
| ENSG00000099194 | SCD | -0.560655865 | 1.43E-07 |
| ENSG00000100030 | MAPK1 | -0.07889878 | 0.19869661 |
| ENSG00000100253 | MIOX | 3.947028402 | 1.11E-62 |
| ENSG00000100292 | HMOX1 | -0.779425146 | 1.35E-11 |
| ENSG00000100644 | HIF1A | 0.737310385 | 1.26E-19 |
| ENSG00000102125 | TAFAZZIN | 0.534743302 | 3.20E-18 |
| ENSG00000102882 | MAPK3 | -0.615020491 | 9.38E-26 |
| ENSG00000103222 | ABCC1 | 1.349541924 | 4.48E-26 |
| ENSG00000104412 | EMC2 | 0.226318981 | 1.17E-05 |
| ENSG00000105281 | SLC1A5 | 1.089661488 | 3.82E-38 |
| ENSG00000105974 | CAV1 | -3.322550378 | 3.61E-141 |
| ENSG00000106211 | HSPB1 | 1.574095485 | 4.94E-43 |
| ENSG00000106327 | TFR2 | 3.156059676 | 1.15E-124 |
| ENSG00000106799 | TGFBR1 | -0.059168084 | 0.497858606 |
| ENSG00000107159 | CA9 | 5.780263819 | 2.06E-145 |
| ENSG00000107643 | MAPK8 | 0.081938824 | 0.134939749 |
| ENSG00000108839 | ALOX12 | 1.386462762 | 6.87E-18 |
| ENSG00000110218 | PANX1 | 0.512863887 | 2.33E-14 |
| ENSG00000110619 | CARS1 | 0.530055931 | 1.35E-29 |
| ENSG00000111371 | SLC38A1 | 0.989568477 | 4.97E-26 |
| ENSG00000111537 | IFNG | 0.947316558 | 7.22E-07 |
| ENSG00000111684 | LPCAT3 | -0.596041983 | 4.90E-15 |
| ENSG00000112062 | MAPK14 | 0.021438013 | 0.677851691 |
| ENSG00000116016 | EPAS1 | -2.979095759 | 4.46E-211 |
| ENSG00000116044 | NFE2L2 | 0.307596151 | 0.002591739 |
| ENSG00000116171 | SCP2 | -0.360974837 | 8.39E-10 |
| ENSG00000117592 | PRDX6 | 0.224565068 | 0.000259994 |
| ENSG00000118503 | TNFAIP3 | -0.992567983 | 7.50E-19 |
| ENSG00000118513 | MYB | 0.790301702 | 2.64E-06 |
| ENSG00000119969 | HELLS | 2.815493822 | 2.48E-182 |
| ENSG00000120053 | GOT1 | 0.888385922 | 3.47E-31 |
| ENSG00000121879 | PIK3CA | 0.317824655 | 0.000184409 |
| ENSG00000122025 | FLT3 | -0.713000843 | 9.25E-08 |
| ENSG00000122873 | CISD1 | 0.519340837 | 1.34E-16 |
| ENSG00000123983 | ACSL3 | 0.215569484 | 0.000725001 |
| ENSG00000124762 | CDKN1A | -0.939923688 | 2.54E-25 |
| ENSG00000125144 | MT1G | -0.023005066 | 0.916162362 |
| ENSG00000126581 | BECN1 | 0.118973249 | 0.005863227 |
| ENSG00000128016 | ZFP36 | -2.120412963 | 2.90E-75 |
| ENSG00000128272 | ATF4 | 0.095805052 | 0.140787188 |
| ENSG00000128965 | CHAC1 | 1.363391709 | 3.82E-33 |
| ENSG00000129596 | CDO1 | -2.820783048 | 4.16E-77 |
| ENSG00000130066 | SAT1 | -0.167593115 | 0.058789145 |
| ENSG00000130766 | SESN2 | 0.035003835 | 0.666708173 |
| ENSG00000131979 | GCH1 | -0.010548763 | 0.926969811 |
| ENSG00000132356 | PRKAA1 | 0.134488324 | 0.030433196 |
| ENSG00000133794 | ARNTL | -0.215747227 | 0.002199533 |
| ENSG00000134324 | LPIN1 | -0.020168848 | 0.84165156 |
| ENSG00000134824 | FADS2 | 0.48979706 | 0.000125841 |
| ENSG00000135245 | HILPDA | 1.725489719 | 4.28E-38 |
| ENSG00000135423 | GLS2 | 0.911321156 | 1.72E-12 |
| ENSG00000135503 | ACVR1B | 0.522698286 | 1.91E-14 |
| ENSG00000136003 | ISCU | -0.518536901 | 1.60E-28 |
| ENSG00000136381 | IREB2 | 0.058250488 | 0.314557014 |
| ENSG00000136869 | TLR4 | -1.858156236 | 3.74E-59 |
| ENSG00000136960 | ENPP2 | -1.742418071 | 1.03E-55 |
| ENSG00000138413 | IDH1 | 0.755066115 | 1.49E-19 |
| ENSG00000138449 | SLC40A1 | -0.984088863 | 1.05E-15 |
| ENSG00000139687 | RB1 | -0.207493322 | 0.003967799 |
| ENSG00000140464 | PML | 0.103000885 | 0.185450686 |
| ENSG00000141510 | TP53 | 0.639644602 | 3.07E-11 |
| ENSG00000141867 | BRD4 | 0.231590011 | 0.000180659 |
| ENSG00000142657 | PGD | 1.115974935 | 8.12E-20 |
| ENSG00000144554 | FANCD2 | 1.81593353 | 3.40E-129 |
| ENSG00000144848 | ATG3 | 0.150615431 | 0.005471088 |
| ENSG00000145354 | CISD2 | 0.649650927 | 7.61E-33 |
| ENSG00000146648 | EGFR | 1.047090976 | 6.42E-13 |
| ENSG00000147649 | MTDH | 0.348562853 | 2.65E-10 |
| ENSG00000147889 | CDKN2A | 4.011363446 | 1.38E-89 |
| ENSG00000148516 | ZEB1 | -1.565267998 | 8.19E-74 |
| ENSG00000149311 | ATM | -0.038635249 | 0.65892913 |
| ENSG00000151012 | SLC7A11 | 3.698219786 | 7.15E-89 |
| ENSG00000151632 | AKR1C2 | 5.133348009 | 3.74E-89 |
| ENSG00000154518 | ATP5MC3 | 0.764920893 | 1.03E-24 |
| ENSG00000155066 | PROM2 | 2.962590411 | 1.41E-110 |
| ENSG00000155957 | TMBIM4 | -0.487082388 | 1.36E-09 |
| ENSG00000156273 | BACH1 | -0.31805408 | 1.56E-06 |
| ENSG00000156873 | PHKG2 | 0.699447361 | 3.08E-36 |
| ENSG00000160200 | CBS | 1.387917936 | 1.36E-14 |
| ENSG00000160211 | G6PD | 1.570627247 | 1.07E-31 |
| ENSG00000161011 | SQSTM1 | 0.292197836 | 0.000661994 |
| ENSG00000161016 | RPL8 | 0.754683994 | 1.34E-19 |
| ENSG00000161905 | ALOX15 | -1.127627065 | 2.42E-05 |
| ENSG00000162409 | PRKAA2 | 0.927680098 | 9.55E-12 |
| ENSG00000162772 | ATF3 | -1.794935872 | 6.77E-45 |
| ENSG00000163374 | YY1AP1 | 0.304975479 | 3.93E-12 |
| ENSG00000163930 | BAP1 | 0.069054198 | 0.182440852 |
| ENSG00000165637 | VDAC2 | 0.649436273 | 2.65E-19 |
| ENSG00000167107 | ACSF2 | -0.387339049 | 0.000243109 |
| ENSG00000167468 | GPX4 | 0.156532273 | 0.03181639 |
| ENSG00000167770 | OTUB1 | 0.469738698 | 4.58E-19 |
| ENSG00000167996 | FTH1 | -0.297325373 | 0.000145649 |
| ENSG00000168003 | SLC3A2 | 0.840591575 | 2.84E-22 |
| ENSG00000168610 | STAT3 | -0.422493038 | 4.30E-16 |
| ENSG00000175224 | ATG13 | 0.206715308 | 4.84E-06 |
| ENSG00000176108 | CHMP6 | -0.085174588 | 0.183384335 |
| ENSG00000177119 | ANO6 | -1.03896782 | 2.22E-69 |
| ENSG00000177169 | ULK1 | 0.794638627 | 8.91E-32 |
| ENSG00000179148 | ALOXE3 | 3.951013329 | 8.62E-86 |
| ENSG00000179477 | ALOX12B | 2.301192691 | 1.35E-25 |
| ENSG00000179593 | ALOX15B | -1.48462369 | 1.43E-14 |
| ENSG00000181019 | NQO1 | 2.896888655 | 1.39E-67 |
| ENSG00000185122 | HSF1 | 0.635448044 | 8.65E-26 |
| ENSG00000185338 | SOCS1 | 0.223389042 | 0.061109193 |
| ENSG00000185499 | MUC1 | -0.258586494 | 0.16706452 |
| ENSG00000186575 | NF2 | 0.162082043 | 0.009156103 |
| ENSG00000187134 | AKR1C1 | 3.932623311 | 4.33E-57 |
| ENSG00000189403 | HMGB1 | -0.021684828 | 0.74188346 |
| ENSG00000196139 | AKR1C3 | 2.830290734 | 1.38E-39 |
| ENSG00000196365 | LONP1 | 0.580216318 | 1.20E-21 |
| ENSG00000197122 | SRC | 0.670895549 | 4.41E-27 |
| ENSG00000197251 | LINC00336 | 0.872787073 | 9.15E-05 |
| ENSG00000197548 | ATG7 | -0.485366997 | 6.88E-19 |
| ENSG00000197635 | DPP4 | 0.520935098 | 0.010101218 |
| ENSG00000198793 | MTOR | 0.308940516 | 5.55E-09 |
| ENSG00000233237 | LINC00472 | -1.883830661 | 5.47E-31 |
| ENSG00000244005 | NFS1 | 0.845160971 | 2.95E-64 |
| ENSG00000266412 | NCOA4 | -0.562780646 | 2.91E-21 |
| ENSG00000269858 | EGLN2 | -0.444818767 | 4.67E-11 |

**Supplemental table 3.** Gene coefficients in LASSO models.

| Gene | Coefficient |
| --- | --- |
| VDAC2 | 0.012148011 |
| HSF1 | 0.073790829 |
| ACSL3 | 0.072450509 |
| PANX1 | 0.045064154 |
| FADS2 | 0.009367233 |
| PHKG2 | -0.129900334 |
| GLS2 | -0.086602834 |
| CDKN1A | 0.059691951 |

**Supplemental table** **4.** Differentially expressed 121 FRGs were analyzed by univariate Cox regression analysis.

| Gene | HR | pvalue |
| --- | --- | --- |
| PHKG2 | 0.691 | 0.00082 |
| PANX1 | 1.31 | 0.002 |
| CDKN1A | 1.23 | 0.0022 |
| ACSL3 | 1.29 | 0.0031 |
| GLS2 | 0.59 | 0.0049 |
| VDAC2 | 1.26 | 0.01 |
| HSF1 | 1.25 | 0.013 |
| CAV1 | 1.1 | 0.015 |
| FADS2 | 1.1 | 0.025 |
| HSPA5 | 1.24 | 0.026 |
| ISCU | 0.76 | 0.027 |
| ANO6 | 1.22 | 0.032 |
| MTDH | 1.21 | 0.034 |
| LINC00336 | 0.386 | 0.036 |
| MIOX | 0.842 | 0.04 |
| ALOX15 | 0.924 | 0.043 |
| SRC | 1.18 | 0.05 |
| ULK1 | 1.16 | 0.057 |
| ACSL4 | 1.12 | 0.058 |
| TFR2 | 0.824 | 0.063 |
| FLT3 | 0.707 | 0.073 |
| TP63 | 0.943 | 0.082 |
| SLC3A2 | 1.13 | 0.085 |
| PROM2 | 1.07 | 0.085 |
| SLC7A11 | 1.06 | 0.09 |
| LAMP2 | 0.86 | 0.098 |
| CDO1 | 0.856 | 0.099 |
| CISD2 | 1.18 | 0.13 |
| ALOX12B | 1.14 | 0.13 |
| ALOXE3 | 1.19 | 0.13 |
| SCD | 1.07 | 0.13 |
| BACH1 | 1.14 | 0.14 |
| SLC38A1 | 1.09 | 0.15 |
| TAFAZZIN | 0.874 | 0.15 |
| PGD | 1.06 | 0.16 |
| MAPK3 | 1.15 | 0.17 |
| AIFM2 | 1.1 | 0.18 |
| ARNTL | 0.859 | 0.18 |
| PEBP1 | 0.911 | 0.2 |
| NFE2L2 | 0.919 | 0.22 |
| FANCD2 | 1.13 | 0.22 |
| NCOA4 | 0.902 | 0.23 |
| CA9 | 1.03 | 0.27 |
| GCLC | 1.04 | 0.28 |
| EGFR | 1.04 | 0.3 |
| OTUB1 | 1.12 | 0.32 |
| ZFP36 | 1.05 | 0.32 |
| SCP2 | 0.888 | 0.32 |
| TF | 0.955 | 0.34 |
| TNFAIP3 | 1.05 | 0.34 |
| YY1AP1 | 0.895 | 0.35 |
| GOT1 | 1.07 | 0.37 |
| CARS1 | 1.12 | 0.37 |
| NFS1 | 0.898 | 0.38 |
| EGLN2 | 0.919 | 0.38 |
| G6PD | 1.03 | 0.39 |
| ATF3 | 1.04 | 0.39 |
| ATP5MC3 | 1.07 | 0.41 |
| CISD1 | 1.08 | 0.42 |
| CDKN2A | 1.02 | 0.42 |
| HELLS | 0.937 | 0.42 |
| MAPK9 | 1.09 | 0.43 |
| PRDX6 | 1.07 | 0.45 |
| ATG5 | 1.09 | 0.45 |
| ACVR1B | 0.94 | 0.45 |
| ATG3 | 0.914 | 0.45 |
| ATG13 | 1.1 | 0.46 |
| SLC1A5 | 1.05 | 0.47 |
| NQO1 | 1.02 | 0.47 |
| PRKAA1 | 0.939 | 0.49 |
| LINC00472 | 0.894 | 0.5 |
| KEAP1 | 0.952 | 0.51 |
| RPL8 | 1.04 | 0.52 |
| IFNG | 0.953 | 0.53 |
| AKR1C2 | 1.01 | 0.54 |
| PRKAA2 | 0.953 | 0.54 |
| TP53 | 0.97 | 0.55 |
| ELAVL1 | 1.09 | 0.55 |
| CHAC1 | 1.04 | 0.55 |
| FTH1 | 1.04 | 0.56 |
| IDH1 | 1.04 | 0.57 |
| SLC40A1 | 0.976 | 0.57 |
| CBS | 0.898 | 0.58 |
| ENPP2 | 1.03 | 0.59 |
| ACSF2 | 0.968 | 0.61 |
| LPCAT3 | 0.956 | 0.61 |
| STAT3 | 0.95 | 0.62 |
| ALOX5 | 1.02 | 0.62 |
| BID | 1.04 | 0.63 |
| NF2 | 0.954 | 0.63 |
| FH | 1.04 | 0.67 |
| ALOX12 | 1.03 | 0.67 |
| TFRC | 0.983 | 0.68 |
| CS | 1.05 | 0.68 |
| LONP1 | 0.967 | 0.69 |
| AKR1C1 | 0.992 | 0.69 |
| HMOX1 | 1.02 | 0.69 |
| HSPB1 | 0.983 | 0.71 |
| NOX4 | 1.03 | 0.71 |
| ALOX15B | 0.988 | 0.71 |
| EMC2 | 1.04 | 0.73 |
| BRD4 | 1.03 | 0.75 |
| RB1 | 0.975 | 0.75 |
| ABCC1 | 1.01 | 0.77 |
| BECN1 | 1.04 | 0.77 |
| ZEB1 | 0.979 | 0.79 |
| HIF1A | 0.983 | 0.8 |
| MTOR | 1.03 | 0.81 |
| SQSTM1 | 0.987 | 0.82 |
| AKR1C3 | 0.995 | 0.82 |
| CD44 | 0.988 | 0.82 |
| HILPDA | 1.01 | 0.84 |
| TMBIM4 | 0.983 | 0.84 |
| EPAS1 | 0.99 | 0.86 |
| GPX4 | 1.01 | 0.87 |
| MYB | 0.99 | 0.87 |
| TLR4 | 0.99 | 0.87 |
| DPP4 | 0.998 | 0.96 |
| PIK3CA | 0.997 | 0.97 |
| ULK2 | 1 | 0.98 |
| ATG7 | 0.998 | 0.99 |

**Supplemental table 5.** Gene coefficients in multivariate Cox models.

| GeneID | Gene | Coefficient |
| --- | --- | --- |
| ENSG00000165637 | VDAC2 | 0.110110158 |
| ENSG00000185122 | HSF1 | 0.266492273 |
| ENSG00000123983 | ACSL3 | 0.195199078 |
| ENSG00000110218 | PANX1 | 0.102375917 |
| ENSG00000134824 | FADS2 | 0.088781349 |
| ENSG00000156873 | PHKG2 | -0.297263386 |
| ENSG00000135423 | GLS2 | -0.352073259 |
| ENSG00000124762 | CDKN1A | 0.179423808 |

**Supplemental table 6.** Univariate Cox regression analysis of prognostic and clinical features associated with OS in lung cancer patients.

| Factor | HR | pvalue | HR.low | HR.high |
| --- | --- | --- | --- | --- |
| Risk | 2.92 | 2.60E-10 | 2.09 | 4.07 |
| Age | 1 | 0.49 | 0.992 | 1.02 |
| Gender | 1.22 | 0.12 | 0.951 | 1.56 |
| Diagnosis | 0.832 | 0.13 | 0.656 | 1.05 |
| NewTumor | 2.66 | 5.30E-12 | 2.01 | 3.51 |
| TStage | 2.02 | 2.60E-06 | 1.51 | 2.71 |
| MStage | 1.81 | 0.032 | 1.05 | 3.12 |
| NStage | 1.71 | 1.20E-05 | 1.34 | 2.17 |

**Supplemental table 7.** Correlation between ACSL3 expression and clinical information of lung cancer

| **Characteristics** | **Number of cases** | **ACSL3 expression** | | ***P* value** |
| --- | --- | --- | --- | --- |
|  |  | **Low (n=15)** | **High (n=15)** |  |
| **Gender** | | | | |
| Male | 19 (63.3%) | 10 (66.7%) | 9 (60.0%) | 0.478 |
| Female | 11 (36.7%) | 5 (33.3%) | 6 (40.0%) |  |
| **Age(year)** | | | | |
| ≥60 | 18 (60.0%) | 6 (40.0%) | 12 (80.0%) | *0.029* |
| <60 | 12 (40.0%) | 9 (60.0%) | 3 (20.0%) |  |
| **Subtype** | | | | |
| LUAD | 17 (56.7%) | 6 (40.0%) | 8 (53.3%) | 0.526 |
| LUSC | 13 (43.3%) | 6 (40.0%) | 7 (46.7%) |  |
| **Tumor size (cm)** | | | | |
| ≥5 | 6 (20.0%) | 3 (20.0%) | 3 (20.0%) | 1.000 |
| <5 | 24 (80.0%) | 12 (80.0%) | 12 (80.0%) |  |
| **Tumor invasion depth** | | | | |
| T1-2 | 25 (83.3%) | 12 (80.0%) | 13 (87.0%) | 0.345 |
| T3-4 | 5 (16.7%) | 3 (20.0%) | 2 (13.3%) |  |
| **Lymph node metastasis** | | | | |
| N0 | 18 (60.0%) | 8 (53.3%) | 10 (66.7%) | 0.224 |
| N1-2 | 12 (40.0%) | 7 (46.7%) | 5 (33.3%) |  |
| **TNM stage** | | | | |
| Ⅰ+Ⅱ | 24 (80.0%) | 11 (73.3%) | 13 (87.0%) | 0.075 |
| Ⅲ+Ⅳ | 6 (20.0%) | 4 (26.7%) | 2 (13.3%) |  |
| **Smoking history** | | | | |
| Yes | 13 (43.3%) | 5 (33.3%) | 8 (53.3%) | 0.224 |
| No | 17 (56.7%) | 10 (66.7%) | 7 (46.7%) |  |

**Supplemental table 8.** Correlation between CDKN1A expression and clinical information of lung cancer

| **Characteristics** | **Number of cases** | **CDKN1A expression** | | ***P* value** |
| --- | --- | --- | --- | --- |
|  |  | **Low (n=15)** | **High (n=15)** |  |
| **Gender** | | | | |
| Male | 19 (63.3%) | 8 (53.3%) | 11 (73.3%) | 0.067 |
| Female | 11 (36.7%) | 7 (46.7%) | 4 (26.7%) |  |
| **Age(year)** | | | | |
| ≥60 | 18 (60.0%) | 9 (60.0%) | 9 (60.0%) | 1.000 |
| <60 | 12 (40.0%) | 6 (40.0%) | 6 (40.0%) |  |
| **Subtype** | | | | |
| LUAD | 17 (56.7%) | 10 (66.7%) | 7 (46.7%) | 0.224 |
| LUSC | 13 (43.3%) | 5 (33.3%) | 8 (53.3%) |  |
| **Tumor size (cm)** | | | | |
| ≥5 | 6 (20.0%) | 3 (20.0%) | 3 (20.0%) | 1.000 |
| <5 | 24 (80.0%) | 12 (80.0%) | 12 (80.0%) |  |
| **Tumor invasion depth** | | | | |
| T1-2 | 25 (83.3%) | 12 (80.0%) | 13 (87.0%) | 0.345 |
| T3-4 | 5 (16.7%) | 3 (20.0%) | 2 (13.3%) |  |
| **Lymph node metastasis** | | | | |
| N0 | 18 (60.0%) | 9 (60.0%) | 9 (60.0%) | 1.000 |
| N1-2 | 12 (40.0%) | 6 (40.0%) | 6 (40.0%) |  |
| **TNM stage** | | | | |
| Ⅰ+Ⅱ | 24 (80.0%) | 10 (66.7%) | 14 (93.3%) | *0.000* |
| Ⅲ+Ⅳ | 6 (20.0%) | 5 (33.3%) | 1 (6.7%) |  |
| **Smoking history** | | | | |
| Yes | 13 (43.3%) | 6 (40.0%) | 7 (46.7%) | 0.526 |
| No | 17 (56.7%) | 9 (60.0%) | 8 (53.3%) |  |

**Supplemental table 9.** Correlation between FADS2 expression and clinical information of lung cancer

| **Characteristics** | **Number of cases** | **FADS2 expression** | | ***P* value** |
| --- | --- | --- | --- | --- |
|  |  | **Low (n=15)** | **High (n=15)** |  |
| **Gender** | | | | |
| Male | 19 (63.3%) | 9 (60.0%) | 10 (66.7%) | 0.478 |
| Female | 11 (36.7%) | 6 (40.0%) | 5 (33.3%) |  |
| **Age(year)** | | | | |
| ≥60 | 18 (60.0%) | 8 (53.3%) | 10 (66.7%) | 0.224 |
| <60 | 12 (40.0%) | 7 (46.7%) | 5 (33.3%) |  |
| **Subtype** | | | | |
| LUAD | 17 (56.7%) | 6 (40.0%) | 11 (73.3%) | 0.157 |
| LUSC | 13 (43.3%) | 9 (60.0%) | 4 (26.7%) |  |
| **Tumor size (cm)** | | | | |
| ≥5 | 6 (20.0%) | 4 (26.7%) | 2 (13.3%) | 0.075 |
| <5 | 24 (80.0%) | 11 (73.3%) | 13 (86.7%) |  |
| **Tumor invasion depth** | | | | |
| T1-2 | 25 (83.3%) | 11 (73.3%) | 14 (93.3%) | *0.002* |
| T3-4 | 5 (16.7%) | 4 (26.7%) | 1 (6.7%) |  |
| **Lymph node metastasis** | | | | |
| N0 | 18 (60.0%) | 8 (53.3%) | 10 (66.7%) | 0.224 |
| N1-2 | 12 (40.0%) | 7 (46.7%) | 5 (33.3%) |  |
| **TNM stage** | | | | |
| Ⅰ+Ⅱ | 24 (80.0%) | 11 (73.3%) | 13 (86.7%) | 0.075 |
| Ⅲ+Ⅳ | 6 (20.0%) | 4 (26.7%) | 2 (13.3%) |  |
| **Smoking history** | | | | |
| Yes | 13 (43.3%) | 6 (40.0%) | 7 (46.7%) | 0.526 |
| No | 17 (56.7%) | 9 (60.0%) | 8 (53.3%) |  |

**Supplemental table 10.** Correlation between GLS2 expression and clinical information of lung cancer

| **Characteristics** | **Number of cases** | **GLS2 expression** | | ***P* value** |
| --- | --- | --- | --- | --- |
|  |  | **Low (n=15)** | **High (n=15)** |  |
| **Gender** | | | | |
| Male | 19 (63.3%) | 11 (73.3%) | 8 (53.3%) | 0.067 |
| Female | 11 (36.7%) | 4 (26.7%) | 7 (46.7%) |  |
| **Age(year)** | | | | |
| ≥60 | 18 (60.0%) | 8 (53.3%) | 10 (66.7%) | 0.224 |
| <60 | 12 (40.0%) | 7 (46.7%) | 5 (33.3%) |  |
| **Subtype** | | | | |
| LUAD | 17 (56.7%) | 7 (46.7%) | 10 (66.7%) | 0.224 |
| LUSC | 13 (43.3%) | 8 (53.3%) | 5 (33.3%) |  |
| **Tumor size (cm)** | | | | |
| ≥5 | 6 (20.0%) | 5 (33.3%) | 1 (6.7%) | *0.000* |
| <5 | 24 (80.0%) | 10 (66.7%) | 14 (93.3%) |  |
| **Tumor invasion depth** | | | | |
| T1-2 | 25 (83.3%) | 11 (73.3%) | 14 (93.3%) | *0.002* |
| T3-4 | 5 (16.7%) | 4 (26.7%) | 1 (6.7%) |  |
| **Lymph node metastasis** | | | | |
| N0 | 18 (60.0%) | 7 (46.7%) | 11 (73.3%) | 0.067 |
| N1-2 | 12 (40.0%) | 8 (53.3%) | 4 (26.7%) |  |
| **TNM stage** | | | | |
| Ⅰ+Ⅱ | 24 (80.0%) | 10 (66.7%) | 14 (93.3%) | 0.000 |
| Ⅲ+Ⅳ | 6 (20.0%) | 5 (33.3%) | 1 (6.7%) |  |
| **Smoking history** | | | | |
| Yes | 13 (43.3%) | 6 (40.0%) | 7 (46.7%) | 0.526 |
| No | 17 (56.7%) | 9 (60.0%) | 8 (53.3%) |  |

**Supplemental table 11.** Correlation between HSF1 expression and clinical information of lung cancer

| **Characteristics** | **Number of cases** | **HSF1 expression** | | ***P* value** |
| --- | --- | --- | --- | --- |
|  |  | **Low (n=15)** | **High (n=15)** |  |
| **Gender** | | | | |
| Male | 19 (63.3%) | 10 (66.7%) | 9 (60.0%) | 0.478 |
| Female | 11 (36.7%) | 5 (33.3%) | 6 (40.0%) |  |
| **Age(year)** | | | | |
| ≥60 | 18 (60.0%) | 6 (40.0%) | 13 (80.0%) | *0.029* |
| <60 | 12 (40.0%) | 9 (60.0%) | 3 (20.0%) |  |
| **Subtype** | | | | |
| LUAD | 17 (56.7%) | 5 (33.3%) | 12 (80.0%) | 0.116 |
| LUSC | 13 (43.3%) | 10 (66.7%) | 3 (20.0%) |  |
| **Tumor size (cm)** | | | | |
| ≥5 | 6 (20.0%) | 3 (20.0%) | 3 (20.0%) | 1.000 |
| <5 | 24 (80.0%) | 12 (80.0%) | 12 (80.0%) |  |
| **Tumor invasion depth** | | | | |
| T1-2 | 25 (83.3%) | 13 (86.7%) | 12 (80.0%) | 0.345 |
| T3-4 | 5 (16.7%) | 2 (13.3%) | 3 (20.0%) |  |
| **Lymph node metastasis** | | | | |
| N0 | 18 (60.0%) | 9 (60.0%) | 9 (60.0%) | 1.000 |
| N1-2 | 12 (40.0%) | 6 (40.0%) | 6 (40.0%) |  |
| **TNM stage** | | | | |
| Ⅰ+Ⅱ | 24 (80.0%) | 12 (80.0%) | 11 (73.3%) | 0.075 |
| Ⅲ+Ⅳ | 6 (20.0%) | 2 (13.3%) | 4 (26.7%) |  |
| **Smoking history** | | | | |
| Yes | 13 (43.3%) | 7 (46.7%) | 6 (40.0%) | 0.526 |
| No | 17 (56.7%) | 8 (53.3%) | 9 (60.0%) |  |

**Supplemental table 12.** Correlation between PANX1 expression and clinical information of lung cancer

| **Characteristics** | **Number of cases** | **PANX1 expression** | | ***P* value** |
| --- | --- | --- | --- | --- |
|  |  | **Low (n=16)** | **High (n=14)** |  |
| **Gender** | | | | |
| Male | 19 (63.3%) | 12 (73.3%) | 7 (53.3%) | 0.124 |
| Female | 11 (36.7%) | 4 (28.6%) | 7 (43.8%) |  |
| **Age(year)** | | | | |
| ≥60 | 18 (60.0%) | 8 (43.8%) | 10 (73.3%) | 0.077 |
| <60 | 12 (40.0%) | 8 (57.1%) | 4 (25.0%) |  |
| **Subtype** | | | | |
| LUAD | 17 (567%) | 12 (73.3%) | 5 (40.0%) | 0.124 |
| LUSC | 13 (43.3%) | 4 (28.6%) | 9 (56.2%) |  |
| **Tumor size (cm)** | | | | |
| ≥5 | 6 (20.0%) | 5 (25.0%) | 1 (13.3%) | 0.727 |
| <5 | 24 (80.0%) | 11 (78.6%) | 13 (81.2%) |  |
| **Tumor invasion depth** | | | | |
| T1-2 | 25 (83.3%) | 13 (80.0%) | 12 (81.2%) | 0.208 |
| T3-4 | 5 (16.7%) | 3 (21.4%) | 2 (12.5%) |  |
| **Lymph node metastasis** | | | | |
| N0 | 18 (60.0%) | 9 (57.1%) | 9 (68.8%) | 0.155 |
| N1-2 | 12 (40.0%) | 7 (50.0%) | 5 (31. 2%) |  |
| **TNM stage** | | | | |
| Ⅰ+Ⅱ | 24 (80.0%) | 12 (73.3%) | 13 (81.2%) | *0.034* |
| Ⅲ+Ⅳ | 6 (20.0%) | 4 (28.6%) | 2 (12.5%) |  |
| **Smoking history** | | | | |
| Yes | 13 (43.3%) | 8 (43.8%) | 5 (40.0%) | 0.925 |
| No | 17 (56.7%) | 8 (57.1%) | 9 (56. %2) |  |

**Supplemental table 13.** Correlation between PHKG2 expression and clinical information of lung cancer

| **Characteristics** | **Number of cases** | **PHKG2 expression** | | ***P* value** |
| --- | --- | --- | --- | --- |
|  |  | **Low (n=15)** | **High (n=15)** |  |
| **Gender** | | | | |
| Male | 19 (63.3%) | 10 (68.8%) | 9 (56.2%) | 0.478 |
| Female | 11 (36.7%) | 5 (33.3%) | 6 (40.0%) |  |
| **Age(year)** | | | | |
| ≥60 | 18 (60.0%) | 9 (56.2%) | 9 (56.2%) | 1.000 |
| <60 | 12 (40.0%) | 6 (40.0%) | 6 (40.0%) |  |
| **Subtype** | | | | |
| LUAD | 17 (56.7%) | 7 (43.8%) | 10 (68.8%) | 0.224 |
| LUSC | 13 (43.3%) | 8 (53.3%) | 5 (33.3%) |  |
| **Tumor size (cm)** | | | | |
| ≥5 | 6 (20.0%) | 2 (13.3%) | 4 (28.6%) | 0.075 |
| <5 | 24 (80.0%) | 13 (86.7%) | 11 (73.3%) |  |
| **Tumor invasion depth** | | | | |
| T1-2 | 25 (83.3%) | 14 (93.3%) | 11 (73.3%) | *0.002* |
| T3-4 | 5 (16.7%) | 1 (6.7%) | 4 (26.7%) |  |
| **Lymph node metastasis** | | | | |
| N0 | 18 (60.0%) | 8 (53.3%) | 10 (68.8%) | 0.224 |
| N1-2 | 12 (40.0%) | 7 (46.7%) | 5 (33.3%) |  |
| **TNM stage** | | | | |
| Ⅰ+Ⅱ | 24 (800%) | 13 (86.7%) | 11 (73.3%) | 0.075 |
| Ⅲ+Ⅳ | 6 (20.0%) | 2 (13.3%) | 4 (26.7%) |  |
| **Smoking history** | | | | |
| Yes | 13 (43.3%) | 8 (53.3%) | 5 (33.3%) | 0.224 |
| No | 17 (56.7%) | 7 (46.7%) | 10 (66.7%) |  |

**Supplemental table 14.** Correlation between VDAC2 expression and clinical information of lung cancer

| **Characteristics** | **Number of cases** | **VDAC2 expression** | | ***P* value** |
| --- | --- | --- | --- | --- |
|  |  | **Low (n=15)** | **High (n=15)** |  |
| **Gender** | | | | |
| Male | 19 (63.3%) | 9 (56.2%) | 10 (68.8%) | 0.478 |
| Female | 11 (36.7%) | 6 (40.0%) | 5 (33.3%) |  |
| **Age(year)** | | | | |
| ≥60 | 18 (60.0%) | 8 (53.3%) | 10 (68.8%) | 0.224 |
| <60 | 12 (40.0%) | 7 (46.7%) | 5 (33.3%) |  |
| **Subtype** | | | | |
| LUAD | 17 (56.7%) | 7 (46.7%) | 10 (68.8%) | 0.224 |
| LUSC | 13 (43.3%) | 8 (53.3%) | 5 (33.3%) |  |
| **Tumor size (cm)** | | | | |
| ≥5 | 6 (20.0%) | 4 (26.7%) | 2 (13.3%) | 0.075 |
| <5 | 24 (80.0%) | 11 (73.3%) | 13 (86.7%) |  |
| **Tumor invasion depth** | | | | |
| T1-2 | 25 (83.3%) | 11 (73.3%) | 14 (93.3%) | *0.002* |
| T3-4 | 5 (16.7%) | 4 (26.7%) | 1 (6.7%) |  |
| **Lymph node metastasis** | | | | |
| N0 | 18 (60.0%) | 7 (46.7%) | 11 (73.3%) | 0.067 |
| N1-2 | 12 (40.0%) | 8 (53.3%) | 4 (26.7%) |  |
| **TNM stage** | | | | |
| Ⅰ+Ⅱ | 24 (80.0%) | 11 (73.3%) | 13 (86.7%) | 0.075 |
| Ⅲ+Ⅳ | 6 (20.0%) | 4 (26.7%) | 2 (13.3%) |  |
| **Smoking history** | | | | |
| Yes | 13 (43.3%) | 8 (53.3%) | 5 (33.3%) | 0.224 |
| No | 17 (56.7%) | 7 (46.7%) | 10 (66.7%) |  |
